# A Prion‐Like Domain in EBV EBNA1 Promotes Phase Separation and Enables SRRM1 Splicing

**DOI:** 10.1002/advs.202501977

**Published:** 2025-07-29

**Authors:** Xiaoyue Zhang, Zhengshuo Li, Run Zheng, Xiang Zheng, Jia Wang, Can Liu, Yangge Wu, Yuqing Wen, Chunlin Ou, Songqing Fan, Chenxiao Xu, Junrui Tian, Qun Yan, Hao Nan, Xiaodong Xu, Hui Wang, Qiu Peng, Jian Ma

**Affiliations:** ^1^ Hunan Cancer Hospital and The Affiliated Cancer Hospital of Xiangya School of Medicine Central South University Changsha Hunan 410013 China; ^2^ Cancer Research Institute Xiangya School of Basic Medical Science Central South University Changsha Hunan 410078 China; ^3^ NHC Key Laboratory of Carcinogenesis Key Laboratory of Carcinogenesis and Cancer Invasion of the Chinese Ministry of Education Hunan Key Laboratory of Nonresolving Inflammation and Cancer Hunan Key Laboratory of Translational Radiation Oncology Changsha Hunan 410078 China; ^4^ Department of Pathology The First Affiliated Hospital of Guilin Medical University Guilin Guangxi 541001 China; ^5^ Department of Immunology Changzhi Medical College Changzhi Shanxi 046000 China; ^6^ Department of Pathology Xiangya Hospital Central South University Changsha Hunan 410008 China; ^7^ Department of Pathology the Second Xiangya Hospital Central South University Changsha Hunan 410011 China; ^8^ Department of Clinical Laboratory Xiangya Hospital Central South University Changsha 410008 China; ^9^ College of Life Sciences Northwest A&F University Yangling Shaanxi 712100 China

**Keywords:** alternative splicing, EBV‐encoded nuclear antigen 1, phase separation, prion‐like domain, protein aggregation

## Abstract

Epstein‐Barr virus (EBV) nuclear antigen 1 (EBNA1) is necessary to maintain stability of EBV episomes, EBV replication, and causes host genomic instability and promotes tumor cells survival. Recent studies have shown that viruses utilize liquid–liquid phase separation (LLPS) within host cells to form sub‐cellular compartments known as “virus factories”. Prion‐like domains (PrLDs), which resemble structural domains of low complexity, are shown to drive LLPS in vivo. In the current study, a PrLD is identified in EBNA1 and aggregation of EBNA1 proteins is observed in EBV‐positive tumors. EBNA1 condensate interacting molecules are examined and are found that EBNA1 interacts with the splicing factor SRSF1 to regulate alternative splicing of SRRM1 and promote tumor progression. Deleting the EBNA1 PrLD results in defects in protein aggregation, LLPS, alternative splicing regulation, and nasopharyngeal carcinoma cells proliferation. Targeting the PrLD of EBNA1 inhibits the formation of protein aggregation, promotes alternative splicing of SRRM1, and inhibits the progression of nasopharyngeal carcinoma. Here, we report for the first time that EBNA1, a protein from the human oncogenic virus EBV, is a prion‐like protein, combining algorithm prediction and experimental validation. That implies a possible molecular pathogenic mechanism of EBNA1 in neurodegenerative diseases.

## Graphical abstract

This study discoveries that EBV EBNA1 behaves as a prion‐like protein, verified using cell‐based assays and the *Saccharomyces cerevisiae* Sup35p prion identification system. The prion‐like domain of EBNA1 drives liquid–liquid phase separation. EBNA1 interacts with the splicing factor SRSF1 to regulate the expression of the SRRM1 splicing isoforms, thereby promoting EBV‐associated tumor development.

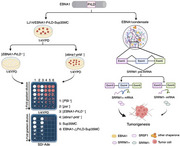

## 1 Introduction

Epstein‐Barr virus (EBV), a human tumor virus, was first identified in African children's Burkitt's lymphoma tissues in 1963.^[^ ^1^, ^2^ ^]^ It establishes lifelong infection in over 90% of global populations.^[^ ^3^, ^4^ ^]^ EBV is tied to multiple tumors like nasopharyngeal carcinoma, Hodgkin's lymphoma, NK/T‐cell lymphomas, and ≈10% of gastric cancers.^[^ ^5^ ^]^ Moreover, it is linked to multiple immune diseases and neurodegenerative diseases, including infectious mononucleosis,^[^ ^6^, ^7^ ^]^ oral hairy leukoplakia,^[^ ^8^ ^]^ systemic lupus erythematosus,^[^ ^9^, ^10^ ^]^ and multiple sclerosis.^[^ ^11^, ^12^ ^]^

EBV‐encoded nuclear antigen 1 (EBNA1) is present in both latent and lytic phases of EBV. It is a defined DNA‐binding protein with multiple functional domains, such as the Gly‐Arg‐rich domain (a.a. 40–64, 325–367), Unique region (a.a. 64–89, 367–379), Gly‐Gly‐Ala repeat (a.a. 90–325), Nuclear localization sequence (a.a. 376–386) and DNA binding domain (a.a. 459–607).^[^ ^13^, ^14^ ^]^ EBNA1 binds to a repeat sequence at EBV DNA's origin of replication (OriP), acting as a transcriptional enhancer to activate viral Cp and LMP gene promoters, playing a part in inducing EBNA and LMP family gene transcription during latent infections.^[^ ^15^, ^16^ ^]^ Additionally, some studies indicate that EBNA1 boosts the malignant progression of multiple cancers.^[^ ^17^ ^]^

Prions are a class of proteins capable of adopting multiple conformations with distinct aggregation propensities, which can undergo conformational transitions to generate new prions.^[^ ^18^, ^19^, ^20^, ^21^ ^]^ Prionogenicity refers to the ability of a protein to form a prion, which are characterized by their capacity to transmit aggregated folding structures.^[^ ^22^, ^23^, ^24^ ^]^ The normal cellular prion protein (PrP^C^) is a cell‐surface glycoprotein widely expressed in neurons, glia, and other tissues. Its physiological roles include the physiology of the central nervous system, resistance to various types of stresses, cell fate and differentiation, cell adhesion, and cell signaling.^[^ ^25^ ^]^ The prion protein (PrP) converts from its normal form PrP^C^ to the pathogenic form PrP^Sc^. These prion diseases have an impact on multiple species. In mammals, the prion protein PrP induces the formation of amyloid plaques in the central nervous system, leading to neurodegenerative diseases.^[^ ^23^, ^24^ ^]^ The most common human prion diseases include Creutzfeldt‐Jakob disease,^[^ ^26^ ^]^ Fatal familial insomnia^[^ ^27^ ^]^ and Gerstmann–Sträussler–Scheinker disease.^[^ ^28^ ^]^ PrP^Sc^ itself can lead to the occurrence of a group of fatal neurodegenerative diseases known as transmissible spongiform encephalopathies or prion diseases.^[^ ^29^ ^]^ Animal prion diseases comprise scrapie in sheep, chronic wasting disease, transmissible mink encephalopathy, chronic wasting disease in deer, and bovine spongiform encephalopathy, as well as other animal prion diseases such as transmissible squirrel encephalopathy and transmissible deer encephalopathy.^[^ ^30^ ^]^ Common features of these prion diseases include progressive neurodegeneration, spongiform encephalopathy, neuronal loss, gliosis, and PrP^Sc^ deposition.

Prion‐like domains are a class of allosteric protein regions, typically composed of arginine‐rich and glutamine‐rich amino acid sequences, functioning as low‐complexity domains that can drive liquid–liquid phase separation in vivo and render proteins infectious.^[^ ^31^, ^32^ ^]^ George Tetz et al. predicted the distribution and functions of prion‐like proteins in eukaryotic viruses using the PLAAC and PAPA prion prediction algorithms. They detected 2679 unique putative prion‐like domains within 2 742 160 publicly available viral protein sequences.^[^ ^18^ ^]^ However, the functions of these prion‐like domain in viral proteins remain unclear. In 2019, Hao Nan et al. identified LEF‐10, a baculovirus protein, as a prion that suppresses late gene expression through state transmission, using the *Saccharomyces cerevisiae* Sup35p prion identification system and cell‐based assays. These discoveries extend the prion research timeline from animals,^[^ ^30^ ^]^ plants,^[^ ^33^ ^]^ fungi,^[^ ^34^ ^]^ bacteria,^[^ ^35^ ^]^ to the final life form‐ viruses.^[^ ^18^, ^21^ ^]^ Studies show that the formation of droplets with prion‐like domains via phase separation is related to protein aggregation in neuronal cells.^[^ ^36^, ^37^ ^]^ Among the four major proteins (α‐synuclein,^[^ ^38^ ^]^ FUS,^[^ ^39^ ^]^ Tau protein,^[^ ^40^ ^]^ and TDP‐43^[^ ^41^ ^]^), these proteins aggregate in different diseases and have each been shown to undergo phase separation. Liquid–liquid phase separation may contribute to the formation of initial intracellular protein aggregates, which may propagate from cell to cell and act as “seeds” to induce further aggregation.

Alternative splicing is crucial for mRNA maturation and enhances RNA and protein diversity.^[^ ^42^ ^]^ SRRM1 (also known as SRm160), a 160 kDa Ser‐Arg (SR)‐associated nuclear matrix protein, serves as a coactivator for both constitutive splicing and exon enhancer‐dependent splicing.^[^ ^43^, ^44^ ^]^ The SRm160/300 complex binds to the splicing complex and facilitates splicing by interacting with SR family proteins.^[^ ^45^ ^]^ However, its role in tumorigenesis remains unclear.

In our previous study, we reported for the first time that the EBV‐encoded nuclear proteins EBNA2 and EBNALP form nuclear speckles and liquid‐like condensates in the cell nucleus, with their IDRs and specific proline residues being necessary for phase separation.^[^ ^46^, ^47^ ^]^ Here, we found that EBNA1 has a prion‐like domain (a.a. 89–350) with glycine and alanine repeats. The aggregates of the EBNA1 proteins are identified in EBV‐positive tumor tissues and are physiologically located in the nucleus. In addition, the prion‐like domain is a prerequisite for the ability of EBNA1 to undergo phase transition. Deletion of the prion domain of EBNA1 inhibits protein aggregation and nasopharyngeal carcinoma cell proliferation. Furthermore, EBNA1 interacts with the splicing factor SRSF1 to regulate the alternative splicing of SRRM1 and promote tumor development in vitro and in vivo. We demonstrated that targeting the prion‐like domain of EBNA1 inhibits protein aggregates formation, promots SRRM1 alternative splicing and inhibits nasopharyngeal cancer progression.

## 2 Results

### 2.1 Identification of a Prion‐Like Domain and Protein Aggregation Properties in EBV EBNA1

**Figure 1:**
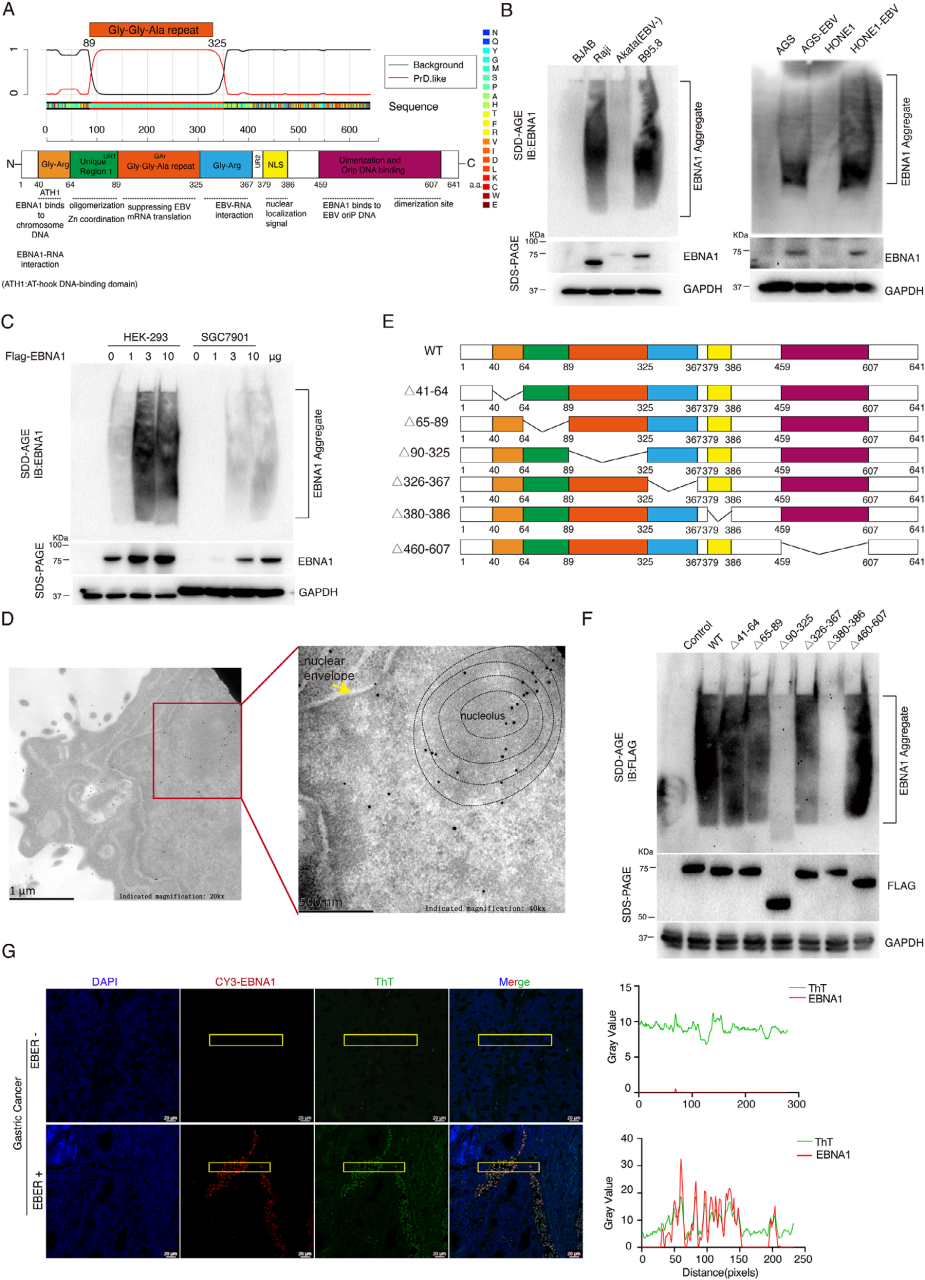
EBV EBNA1 possesses a prion‐like domain and exhibits protein aggregation properties. A) Prediction of prion‐like domains of EBNA1 based on its amino acid positions and sequences was performed using the PLAAC database (http://plaac.wi.mit.edu/). The red line represents the prediction of the prion‐like domain, and if the red line is above the black line, it represents a prion‐like region. The EBNA1 prion‐like region was compared with the EBNA1 amino acid structural domain. B) The SDD‐AGE and SDS‐PAGE assays were performed to detect the expression of endogenous EBNA1 protein aggregates and monomers in multiple cell lines: BJAB (EBV‐negative), Raji (EBV‐positive), Akata (EBV‐negative), B95.8 (EBV‐positive), AGS (EBV‐negative), AGS‐EBV (EBV‐positive), HONE1(EBV‐negative) and HONE‐EBV (EBV‐positive). C) HEK‐293 and SGC7901 cells were transfected with the Flag‐tagged EBNA1 plasmid for 48 h. The expression of exogenous EBNA1 proteins was detected by SDS‐AGE and SDS‐PAGE. D) HEK‐293 cells were transfected with Flag‐tagged EBNA1 plasmid for 48 h, and the cell precipitates were collected and fixed. The ultrathin sections (around 50–70 nm) were prepared and placed on a 200‐mesh gold mesh. They were incubated with anti‐EBNA1 antibody at 4 °C overnight and goat anti‐mouse IgM/Gold (10 nm) secondary antibody for 1 h at room temperature. The subcellular localization of EBNA1 protein aggregates was detected by transmission electron microscopy. The yellow arrow indicates the cell's nuclear membrane. E) Schematic representation of a series of truncated vectors with Flag‐tagged EBNA1 structural domains. F) HEK‐293 cells were transfected with Flag‐tagged EBNA1 truncated plasmids for 48 h, separately. The expression of EBNA1 protein aggregates was detected by SDD‐AGE analysis. G) Gastric cancer tissue samples (either EBER+ or EBER‐) were collected for immunofluorescence and thioflavin T (final concentration of 400 mM) staining. Co‐localization of Cy3‐labeled EBNA1 with thioflavin T green fluorescence was observed by fluorescence microscopy. Cy3 labels EBNA1; Thioflavin T: green fluorescence, labels amyloid aggregates; DAPI: labels nuclei. Scale bar: 20 µm.

We used the PLAAC website to predict protein disorder and amino acids composition similarity to known yeast prions.^[^ ^48^ ^]^ EBV EBNA1 contains a disordered region at the Gly‐Gly‐Ala repeat domain (a.a.90‐325) with prion‐like properties (**Figure** **1**A). For comparison, we also validated the amino acid sequences of the EBNA family proteins (EBNA2, EBNALP, EBNA3A, EBNA3B, EBNA3C), and only EBNA3C has a prion‐like domain at a.a.740‐793 (Figure S1A, Supporting Information).

To investigate EBNA1 protein aggregation under physiological conditions, proteins from EBV‐positive cells (Raji, B95.8, AGS‐EBV and HONE1‐EBV) and EBV‐negative cells (BJAB, Akata and AGS) were collected and examined by semi‐denaturing agarose gel electrophoresis (SDD‐AGE),^[^ ^49^, ^50^ ^]^ which is a tool to identify amyloid‐like protein aggregates. The endogenous EBNA1 forms protein aggregates in EBV‐positive cells (Figure 1B). Exogenous Flag‐tagged EBNA1 plasmids transfected into HEK‐293 and SGC7901 cells also led to aggregation under physiological conditions (Figure 1C). After transfecting Flag‐tagged EBNAs plasmids (EBNA1, EBNA2, EBNALP, EBNA3A, EBNA3B and EBNA3C) into HEK‐293 cells, we found that only EBNA1 and EBNA3C showed protein aggregation, however, the aggregation phenomenon of EBNA3C was much weaker than that of EBNA1 (Figure S1B, Supporting Information).

We next used immunoelectron microscopy to explore EBNA1 protein aggregates’ sub‐cellular localization. Flag‐tagged EBNA1 plasmids were transfected into HEK‐293 cells; electron micrographs revealed a large number of 10 nm gold particles (i.e., Flag‐tagged EBNA1 proteins) were distributed in the nucleus showing aggregation (Figure 1D). We further investigate whether the EBNA1 structural domain affects protein aggregate formation. A series of EBNA1 structural domain truncation plasmids (including EBNA1△41–64, △65–89, △90–325, △326–367, △380–386, and △460–607) were transfected into HEK‐293 cells, respectively, and cell lysates were harvested for SDD‐AGE analysis. EBNA1 protein aggregates disappeared after deleting amino acids 90–325 or amino acids 380–386 (Figure 1E,F). The absence of the nuclear localization signal domains (a.a. 380–386) caused the disappearance of protein aggregation because EBNA1 cannot enter the nucleus normally. Expression of EBNA1 promoted the proliferation of nasopharyngeal carcinoma cell HONE1 (Figure S1C, Supporting Information), whereas deletion of EBNA1 prion‐like domain abolished this ability (Figure S1D,E, Supporting Information).

To examine whether EBNA1 protein aggregates exist in EBV‐positive tumor tissues, we stained the clinical tumor tissues using thioflavin T, a benzothiazole dye binding to amyloid with enhanced fluorescence signal.^[^ ^51^ ^]^ In EBER (+) gastric cancer tissues, Cy3‐labeled EBNA1 co‐localized with thioflavin T green fluorescence in the nucleus, while not in the EBER (−) tissues. This finding indicates EBNA1 protein aggregation in the EBER (+) gastric cancer tissues (Figure 1G). Similarly, EBNA1 protein aggregation was also present in EBER (+) lymphoma (Figure S1F, Supporting Information) and EBV‐positive nasopharyngeal cancer tissues (Figure S1G, Supporting Information).

### 2.2 EBNA1 Behaves as a Prion‐Forming Protein in the Yeast Sup35‐Based Prion Assay

To characterize the prion properties of EBNA1‐PrLD, we employed the *Saccharomyces cerevisiae* Sup35 prion assay system, a well‐validated prion phenotype detection method based on the characterized prion properties of the yeast translation termination factor Sup35.^[^ ^22^, ^52^ ^]^ Sup35 consists of an N‐terminal prion domain (N), a highly charged middle domain (M), and a C‐terminal domain (C) that provides translation termination function. Since Sup35p is an essential protein, we utilized a *Saccharomyces cerevisiae* strain LJ14 (*MATa ade1‐14 trp1‐289 his3Δ‐200 ura3‐52 leu2‐3112 SUP35::loxP p[SUP35‐URA3*] [*PSI* ^+^]),^[^ ^53^ ^]^ in which the chromosomal Sup35 deletion is complemented by a plasmid expressing Sup35. When these cells were transformed with a PrLD‐Sup35MC expression plasmid, the URA3 marker on the covering Sup35 plasmid enabled negative selection on medium containing 5‐FOA (termed plasmid shuffle). Yeast ade1‐14 cells expressing EBNA1‐PrLD‐Sup35MC failed to grow on Ura‐deficient medium (SD/‐Ura), but could grow on His‐deficient medium(SD/‐His) (Figure S2A,B, Supporting Information).The resulting strains contained the EBNA1‐PrLD‐Sup35MC fusion protein as the sole source of functional Sup35. The ability of each strain to form heritable [*PSI* ^+^] states was tested using an ADE1 allele with a premature stop codon. Readthrough of this allele in [*PSI* ^+^] cells produces two readily monitorable phenotypes: the ability to grow on adenine‐deficient medium (SD/‐Ade) and white colony color on complete medium (1/4YPD) due to restoration of the adenine biosynthesis pathway, which prevents accumulation of red by‐products in [*psi* ^−^] cells.^[^ ^21^, ^22^ ^]^

**Figure 2:**
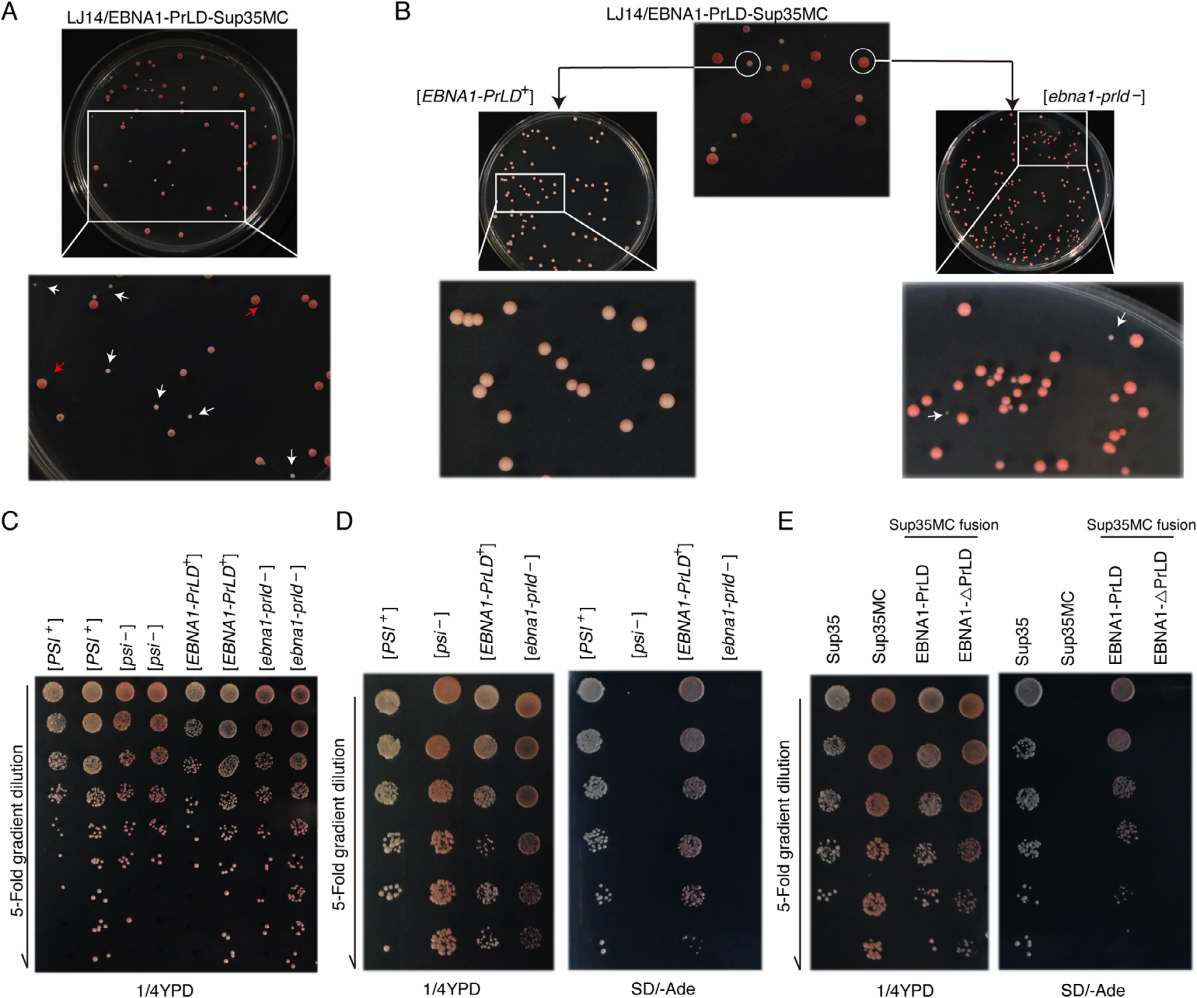
The yeast Sup35‐based prion assay is used to detect EBNA1 prion‐like behavior.A–D) The yeast cells expressing EBNA1‐PrLD‐Sup35MC plated on complete medium (1/4 YPD). [*EBNA1‐PrLD* ^+^] was phenotypically similar to [*PSI* ^+^], producing white colonies, which could be distinguished from [*ebna1‐prld* ^−^] strains by red pigments accumulated through the adenine biosynthesis pathway (A, B top). Both phenotypes were stably maintained during repeated cell passaging (B, middle). [*ebna1‐prld* ^−^] strains spontaneously generated[*EBNA1‐PrLD* ^+^] clones at a low frequency (B, bottom). White arrows indicate [*EBNA1‐PrLD* ^+^] clones, and red arrows indicate [*ebna1‐prld* ^−^] clones. 5‐Fold serial dilutions of [*PSI* ^+^], [*psi* ^−^], [*EBNA1‐PrLD* ^+^], and [*ebna1‐prld* ^−^] clone were individually spotted onto complete (1/4YPD) medium (C) and adenine‐deficient medium (SD/‐Ade)(D, right). E) Comparable characteristics of EBNA1‐PrLD‐Sup35MC, EBNA1‐△PrLD‐Sup35MC and full‐length Sup35 in yeast. Fivefold serial dilutions of these clones were individually spotted onto complete (1/4YPD) medium and adenine‐deficient medium (SD/‐Ade).

We replaced the prion domain (PrLD) of Sup35 with EBNA1‐PrLD to generate the EBNA1‐PrLD‐Sup35MC fusion protein. Experiments showed that similar to yeast containing wild‐type Sup35, yeast ade1‐14 cells expressing EBNA1‐PrLD‐Sup35MC plated on complete medium (1/4 YPD) had [*EBNA‐PrLD* ^+^] strains forming white colonies, which were distinguishable from [*ebna1‐prld* ^−^] strains by red pigment accumulation via the adenine biosynthesis pathway (**Figure** **2**A,B). Both phenotypes were maintained during cell proliferation. In [*ebna1‐prld* ^−^] cells, replacement of Sup35 with the EBNA1‐PrLD‐Sup35MC fusion protein generated colonies with a red, Ade− phenotype, indicating that the EBNA1‐PrLD‐Sup35MC fusion protein efficiently terminates translation at the premature UGA stop codon in the ade1‐14 allele. Conversely, similar to [*PSI* ^+^] strains, [*EBNA1‐PrLD* ^+^] cells expressing the chimeric EBNA1‐PrLD‐Sup35MC protein showed significantly reduced termination efficiency, leading to readthrough of the premature stop codon in ade1‐14 and the emergence of a white Ade+ phenotype identical to that seen in [*PSI* ^+^] cells (Figure 2C,D). In contrast, EBNA1‐△PrLD‐Sup35MC chimeric proteins lacking the PrLD exhibited a [*psi* ^−^]‐like phenotype, confirming that the observed [*EBNA1‐PrLD* ^+^] phenotype is dependent on the PrLD of EBNA1 (Figure 2E). These results support our speculation that EBNA1 is a prion‐like protein.

### 2.3 The Prion‐Like Domain is Essential for EBNA1 Phase Separation

**Figure 3:**
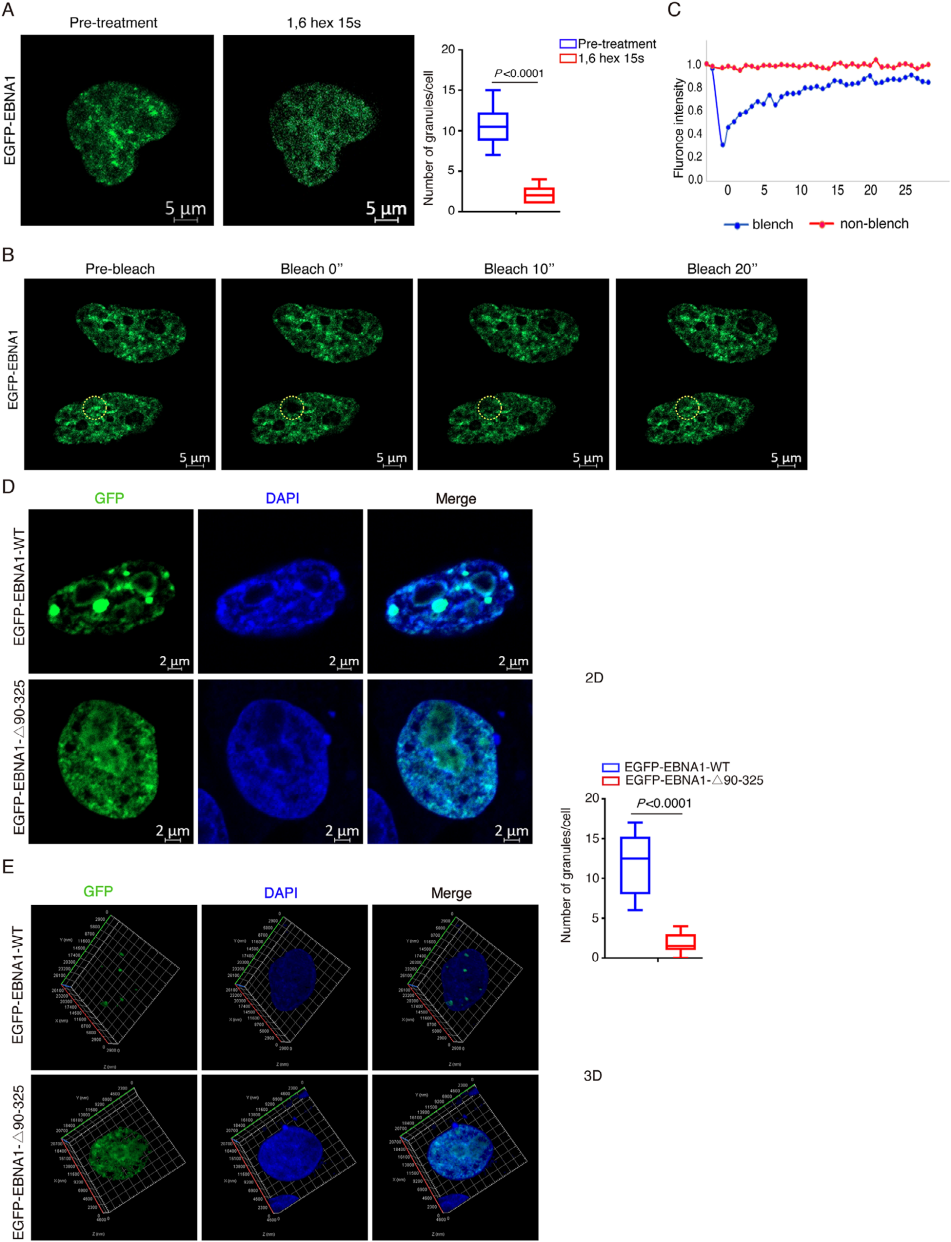
The prion‐like domain of EBNA1 drives phase separation. A) HEK‐293 cells transfected with EGFP‐EBNA1 plasmid were treated with 3% of 1,6‐hexanediol for 15 s. The upper panel is a representative image of the spot changes of EGFP‐EBNA1. B,C) HEK‐293 cells were transfected with EGFP‐EBNA1 plasmid for fluorescence recovery after photobleaching assay. A representative image is shown. The yellow circle highlights the spot undergoing targeted bleaching (B). Quantification of FRAP fluorescence intensity data of EGFP‐EBNA1 spots, normalized to 100% by maximum fluorescence intensity (C). D,E) HEK‐293 cells were transfected with EGFP‐EBNA1‐WT or EGFP‐EBNA1‐△90‐325 truncated plasmid for immunofluorescence imaging. Laser confocal detection of EBNA1 liquid condensate formation in the nucleus. 2D fluorescence images are shown in (D), and 3D fluorescence images are shown in (E).

Proteins containing prion‐like domains exhibit liquid–liquid phase separation, where the prion‐like domain provides conditions that facilitate this phenomenon.^[^ ^32^, ^54^ ^]^ We hypothesize that EBNA1 forms liquid‐like condensates in the nucleus. To investigate this further, we treated HEK‐293 cells transfected with the EGFP‐EBNA1 plasmid using 1,6‐hexanediol, which disrupts liquid‐like condensates by disturbing the hydrophobic interactions, and observed a significant reduction in the number of EGFP‐EBNA1 puncta in the nucleus (**Figure** **3**A). We utilized fluorescence bleaching recovery experiments^[^ ^55^, ^56^ ^]^ to assess whether the EBNA1 nuclear puncta have liquid condensate dynamic properties. After photobleaching, EGFP‐EBNA1 punctate regained fluorescence in seconds (Figure 3B,C), showing EBNA1 forms spots in the nucleus with dynamic liquid condensate properties. We transfected HEK‐293 cells with the EGFP‐tagged EBNA1 and EBNA1‐Δ90‐325 truncated plasmids, and subsequently analyzed their expression and localization via laser scanning confocal microscopy. Our observations from both 2D and 3D confocal images revealed that EGFP‐tagged EBNA1 formed liquid‐like condensates within the nucleus, manifesting as numerous variable‐sized punctate. In contrast, the nuclei of cells transfected with EBNA1‐Δ90‐325 did not show such punctate (Figure 3D,E).

### 2.4 Splicing Factor SRSF1 Interacts with EBNA1

**Figure 4:**
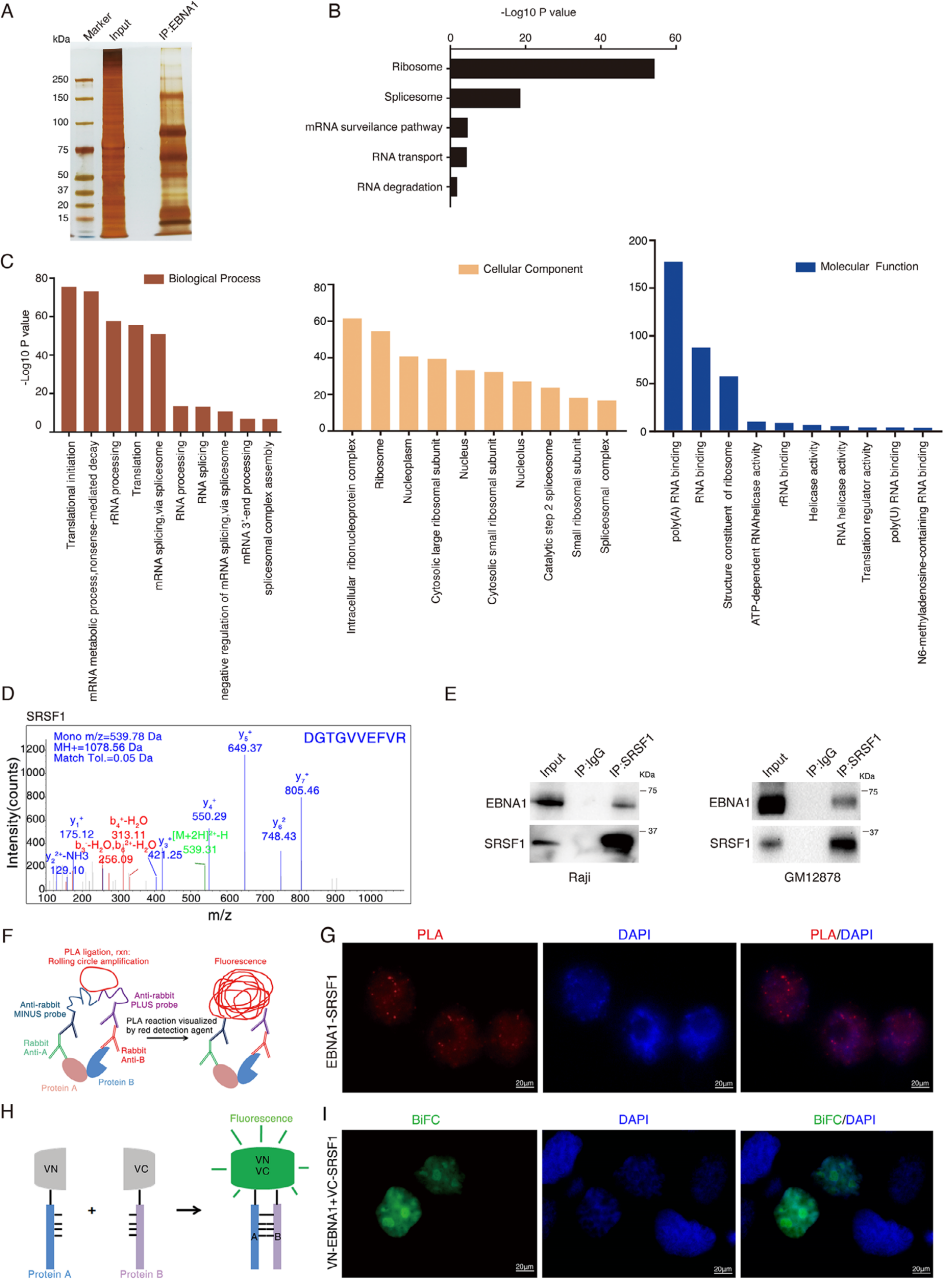
EBNA1 interacts with components of the splicing machinery. A) Raji cell protein lysates were collected and immunoprecipitated with anti‐EBNA1 antibody, and the precipitated proteins were analyzed by SDS‐PAGE assay and mass spectrometry. B,C) KEGG (B) and GO enrichment analyses (C) were performed to detect the major signaling pathways enriched for EBNA1 interacting proteins using the mass spectrometry data. D) Immunoprecipitation combined with mass spectrometry analysis showed the peak of the SRSF1 peptide, indicating that SRSF1 was present in the EBNA1 precipitate complex. E) Immunoprecipitations were performed with anti‐EBNA1, anti‐SRSF1 antibody or normal anti‐IgG antibody in Raji cells or GM12878 cells. F,G) Proximity ligation assay was performed to detect the interaction between EBNA1 and SRSF1 in Raji cells. Model diagram of Proximity Ligation Assay (F). Fluorescence microscopy was performed to observe the fluorescence co‐localization of EBNA1 with SRSF1 (G). H,I) HEK‐293 cells were transfected with pBiFC‐VC‐SRSF1 and pBiFC‐VN‐EBNA1 plasmids for 48 h, and then a bimolecular fluorescence complementation assay was performed. Model diagram of Bimolecular fluorescence complementation (H). The fluorescence expression of EBNA1 and SRSF1 was detected by fluorescence microscopy (I). Scale bar: 20 µm.

To identify EBNA1‐interacting components in the nuclear puncta, we used mass spectrometry to analyze the EBNA1 coimmunoprecipitates in Raji cells, and found that 295 cellular proteins potentially interacted with EBNA1 (**Figure** **4**A). Notably, these proteins were predominantly enriched in ribosomes and RNA splicing, as determined by Kyoto Encyclopedia of Genes and Genomes (KEGG) pathway enrichment analysis (Figure 4B). Gene Ontology (GO) enrichment analysis indicated that the majority of the interacting proteins were enriched in the RNA splicing signaling pathway or RNA spliceosome (Figure 4C). The Metascape^[^ ^57^ ^]^ (http://metascape.org/) also revealed significant interactions, primarily involving mRNA splicing, and mRNA metabolism (Molecular Complex Detection, MCODE) (Figure S3, Supporting Information). These findings indicate EBNA1 potentially modulates cellular biological processes by engaging with spliceosome components.

Among 295 EBNA1‐interacted proteins, splicing factors SRSF1's peptide patterns have a considerable high score, indicating a potential interaction between EBNA1 and SRSF1 (Figure 4D). SRSF1, a prominent member of the SR protein family, plays a crucial role in spliceosome maturation.^[^ ^58^, ^59^ ^]^ To validate the interaction between EBNA1 and SRSF1, immunoprecipitation experiments were conducted, revealing the interaction of endogenous SRSF1 with EBNA1 in EBV‐positive cells (Figure 4E). Moreover, proximity ligation assay (PLA) further confirmed this interaction, and showed their co‐localization in the nucleus (Figure 4F,G). We established a bimolecular fluorescence complementation (BiFC) system^[^ ^60^ ^]^ for visualizing and analyzing the interaction between EBNA1 and SRSF1. Co‐expressing pBiFC‐VN‐EBNA1 and pBiFC‐VC‐SRSF1 (two fragments containing either the GFP N‐terminal, VN or GFP C‐terminal, VC) in cells led to an intense BiFC signal (green fluorescence signal in the nucleus), confirming the interaction between EBNA1 and SRSF1 (Figure 4H,I).

### 2.5 EBNA1‐Induced Alternative Splicing of SRRM1 is Regulated by its Prion‐Like Domain

**Figure 5:**
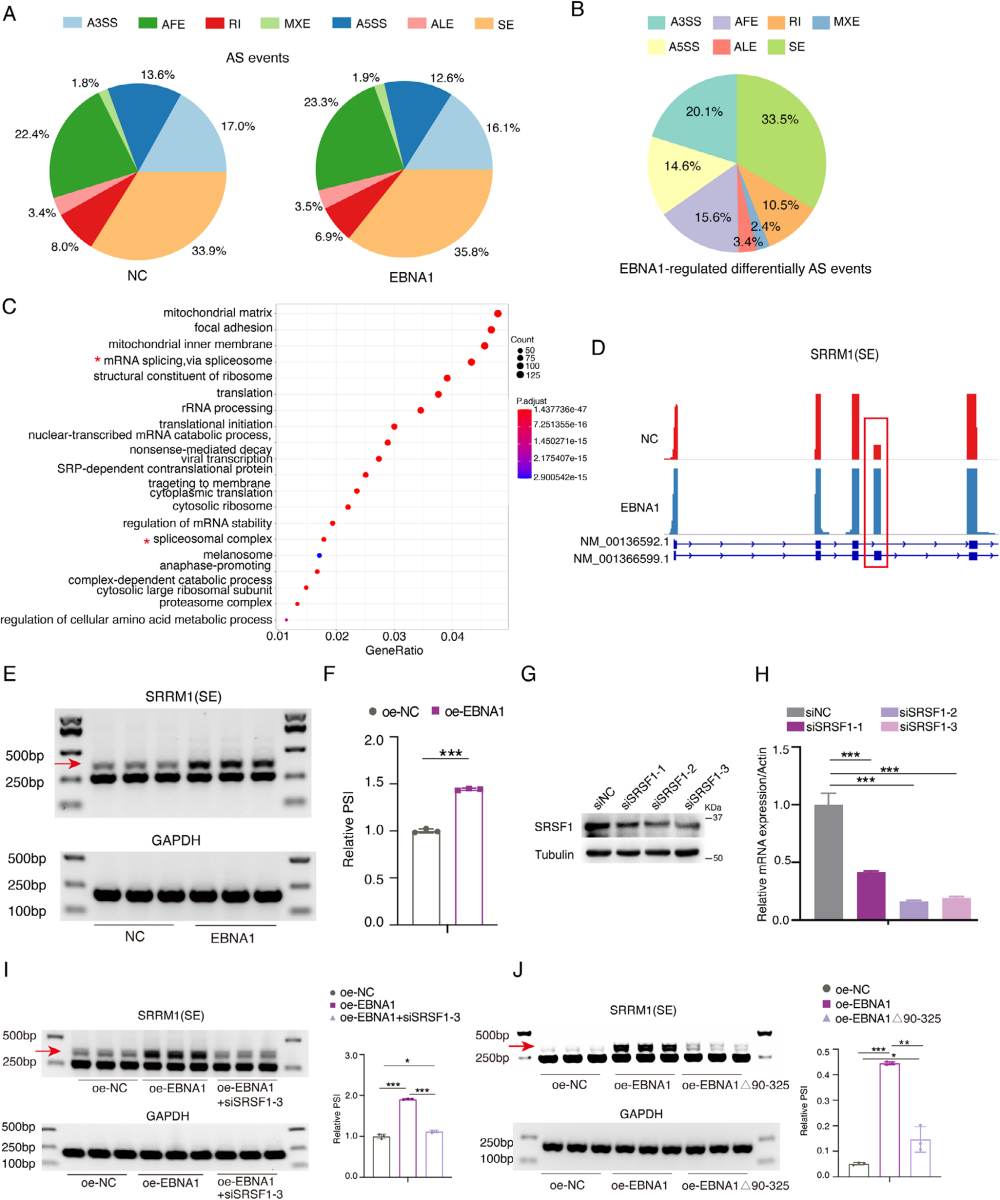
EBNA1 modulates cellular alternative splicing events and regulates SRRM1 exon skipping. A) HEK‐293 cells were transfected with either the EBNA1 expression vector or the empty vector (NC) for 48 h. The proportion of alternative splicing events in both cells is revealed by a third‐generation sequencing platform. SE: exon skipping, RI: intron retention, A5SS: alternative 5′ splice sites, A3SS: alternative 3′splice sites, AFE: alternative first exon, ALE: alternative last exon, MXE: mutually exclusive exon. B) The proportion of EBNA1‐regulated differentially alternative splicing events (EBNA1 vs NC). C) GO enrichment analysis of the biological functions of EBNA1‐regulated differentially alternative spliced genes. D) Normalized sequencing reads count the relative expression of each exon of the SRRM1 gene. Red bars indicate that EBNA1‐specific isoforms shown with exon arrangement and alternative splicing sites. E) The red arrow marks the position of EBNA1‐regulated SRRM1 alternative splice isoforms. GAPDH was used as a loading control. F) The relative expression of the SRRM1(+) isoform was shown. G,H) HONE1 cells were transfected with the indicated siRNAs for 48 h to detect SRSF1 protein (G) and mRNA expression (H). I) HONE1 cells were transfected with Flag‐EBNA1 with or without siSRSF1‐3 for 48 h. Cells were collected for RT‐PCR to detect SRRM1 alternative splice isoforms. GAPDH was used as a loading control. RT‐qPCR was tested on three biological replicates. J) HONE1 cells transfected with Flag‐EBNA1 or Flag‐EBNA1△90‐325 plasmids for 48 h. Cells were collected for RT‐PCR and agarose gel electrophoresis. Red arrows indicate the positions of SRRM1 alternative splice isoforms. RT‐PCR was tested on three biological replicates. These data are shown as the Mean ± SD. *P < 0.05, **P < 0.01, ***P < 0.001.

The above discovery prompts us to ask whether EBNA1 regulates host cell alternative splicing events via splicing factor interaction. We performed full‐length transcriptome sequencing using a third‐generation sequencing platform, and analyzed differential alternative splicing events and gene expression induced by EBNA1 expression (Figure S4A, Supporting Information; **Figure** **5**A). Comparing to NC (negative control) group, EBNA1 induced 507 significantly differentially alternative splicing (DAS) events. Among them, 33.5% events were exon skipping, 14.6% and 20.1% were 5′and 3′ alternative splicing events, respectively (Figure 5A,B). GO enrichment analysis of DAS genes revealed that these genes were mainly enriched in categories of mitochondrial matrix, mRNA splicing, etc. (Figure 5C). KEGG enrichment analysis revealed these DAS genes were mainly enriched in the categories of neurodegenerative diseases, like amyotrophic lateral sclerosis (ALS), Alzheimer's disease, prion disease, etc. (Figure S4B, Supporting Information). These suggest that EBNA1‐regulated alternative spliced genes may be linked to diseases with amyloid aggregation, aiding understanding the role of EBNA1 in neurodegenerative diseases development. KEGG and GO enrichment analysis were also performed on the EBNA1‐induced differentially expressed genes (DEG). The DEGs were mainly enriched in the metabolism‐related functional categories such as the regulation of MAP kinase activity, galactose metabolism, etc. (Figure S4C, Supporting Information).

To verify EBNA1's function in regulating alternative splicing events, we selected candidate isoforms crucial in exon skipping based on KEGG and GO enrichment analysis results. During isoforms screening, we found EBNA1 might regulate SRRM1 exon 4 skipping. The Percentage Spliced In (PSI) value of SRRM1 exon 4 inclusion splice variants (SRRM1+) were significantly increased by EBNA1 (Figure 5D). Agarose gel electrophoresis and RT‐PCR confirmed that expression of EBNA1 inhibited SRRM1 exon 4 skipping (Figure 5E,F). This indicates EBNA1 can promote SRRM1(+) splice isoform generation. SRRM1 is an SR family protein member with at least one RNA recognition motif and an extensively phosphorylated carboxyl‐terminal structural domain enriched in serine/arginine dipeptides, which function in pre‐mRNA splicing.^[^ ^61^ ^]^

EBNA1's interacting molecules are mostly enriched in mRNA spliceosome's functional category, implying that EBNA1 can interact with spliceosome to regulate SRRM1 alternative splicing as per mass spectrometry results. Therefore, we hypothesize that EBNA1 may interact with SRSF1 to regulate SRRM1 exon skipping event. We inhibited SRSF1 protein and RNA expression levels in HONE1 cells using siRNA (Figure 5G,H). The results showed that inhibition of SRSF1 could promote SRRM1 exon 4 skipping (Figure 5I), suggesting that EBNA1‐mediated SRRM1 (+) splicing isoform production is mediated by SRSF1. We hypothesized that EBNA1 regulates SRRM1 alternative splicing through its prion‐like domain. Agarose gel electrophoresis showed that WT EBNA1 promoted SRRM1 exon 4 inclusion, whereas prion‐like domain deletion reversed this effect (Figure 5J), suggesting the prion‐like domain is responsible for EBNA1's regulation of SRRM1 alternative splicing events.

### 2.6 EBNA1‐Regulated Alternative Splicing of SRRM1 Promotes Malignant Phenotype In Vitro and In Vivo

**Figure 6:**
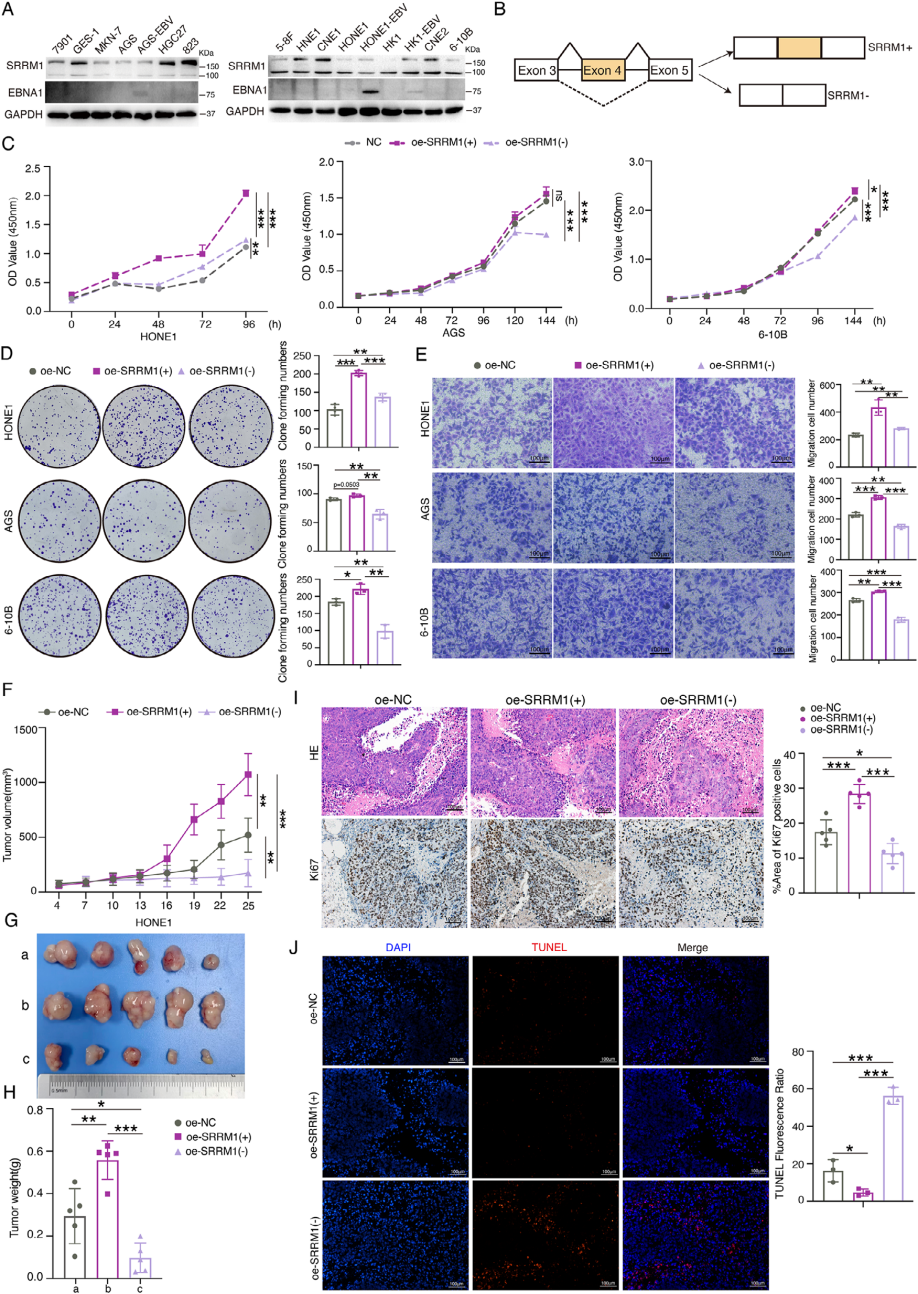
EBNA1‐mediated SRRM1(+) splice isoform facilitates malignant phenotype in tumor cells. A) The protein expression levels of SRRM1 in various gastric and nasopharyngeal carcinoma cells were assayed by Western blotting. B) Patterns of different splice isoforms generated after an exon‐skipping event in SRRM1. C–E) HONE1, AGS, and 6–10B cells were transfected with Flag‐SRRM1 (+) or Flag‐SRRM1 (−) plasmids, respectively. Then, CCK8 (C), Clone formation (D), and Transwell assays (E) were performed to detect tumor cell proliferation and cell migration abilities. The experiments were independently repeated three times, and the results are presented as mean ± SD. F–H) HONE1 cells stably transfected with Flag‐SRRM1(+) or Flag‐SRRM1(−) plasmids were subcutaneously implanted BALB/c nude mice to establish a xenograft growth model. The tumor volume was measured every three days, and growth curves were plotted (F). When the tumor cells grew for 25 days, the mice were euthanized and the tumor tissues were removed. The tumor volumes of each group were recorded (G) and tumor tissues were weighed (H). Each group has 5 mice. I)H&E staining and immunohistochemical analysis of tumor tissue morphology and Ki67 expression levels. J) TUNEL staining was performed to detect the level of apoptosis in tumor tissues of different treatment groups. These data are shown as the mean ± SD. Scale bar: 100 µm.*P < 0.05, **P < 0.01, ***P < 0.001, ns, not significant.

We next investigated the role of SRRM1 in nasopharyngeal and gastric tumorigenesis. We examined SRRM1 protein expression levels in multiple gastric and nasopharyngeal cancer cells by Western blotting. Comparable high levels of SRRM1 were observed in human gastric mucosal cells GES‐1, gastric cancer cells HGC27, BGC‐823, and nasopharyngeal cancer cells HNE1, CNE1, and CNE2 (**Figure** **6**A). Two splice isoforms, namely SRRM1(+) and SRRM1(−), are generated by SRRM1 exon 4 inclusion or skipping (Figure 6B). Expression of SRRM1(+) promoted the proliferation of nasopharyngeal carcinoma cells HONE1 and 6–10B, and had no significant effect on gastric carcinoma cells AGS, comparing to the control group (oe‐NC). However, SRRM1(−) had weaker pro‐proliferative capacity than SRRM1(+) as detected by CCK8 and clone formation assay (Figure 6C,D; Figure S5A, Supporting Information). Cell migration assay indicated SRRM1(+) boosted the migration of AGS, HONE1 and 6–10B cells, whereas SRRM1(−) had the opposite effect (Figure 6E).

To further clarify the function of SRRM1 splice isoforms on tumor progression in vivo, we established a tumor growth model by injecting HONE1 cells stably expressing SRRM1(−) or SRRM1(+) into the nude mice. Overexpression of SRRM1(+) significantly promoted the tumor growth in vivo, whereas SRRM1(−) suppressed it (Figure 6F–H; Figure S5B, Supporting Information). Expression of SRRM1(+) promoted cell proliferation by means of Ki67 staining (Figure 6I) and decreased cell apoptosis by TUNEL staining (Figure 6J), while SRRM1(−) had the opposite effects. These results revealed that EBNA1‐mediated SRRM1 splicing isoforms have different functions, with SRRM1(+) promoting nasopharyngeal carcinoma cell proliferation in vivo and in vitro, while SRRM1(−) functions in the opposite manner.

### 2.7 Targeting the Prion‐Like Domain of EBNA1 Inhibits Protein Aggregate Formation, Promotes SRRM1 Alternative Splicing and Inhibits Nasopharyngeal Carcinoma Progression

**Figure 7:**
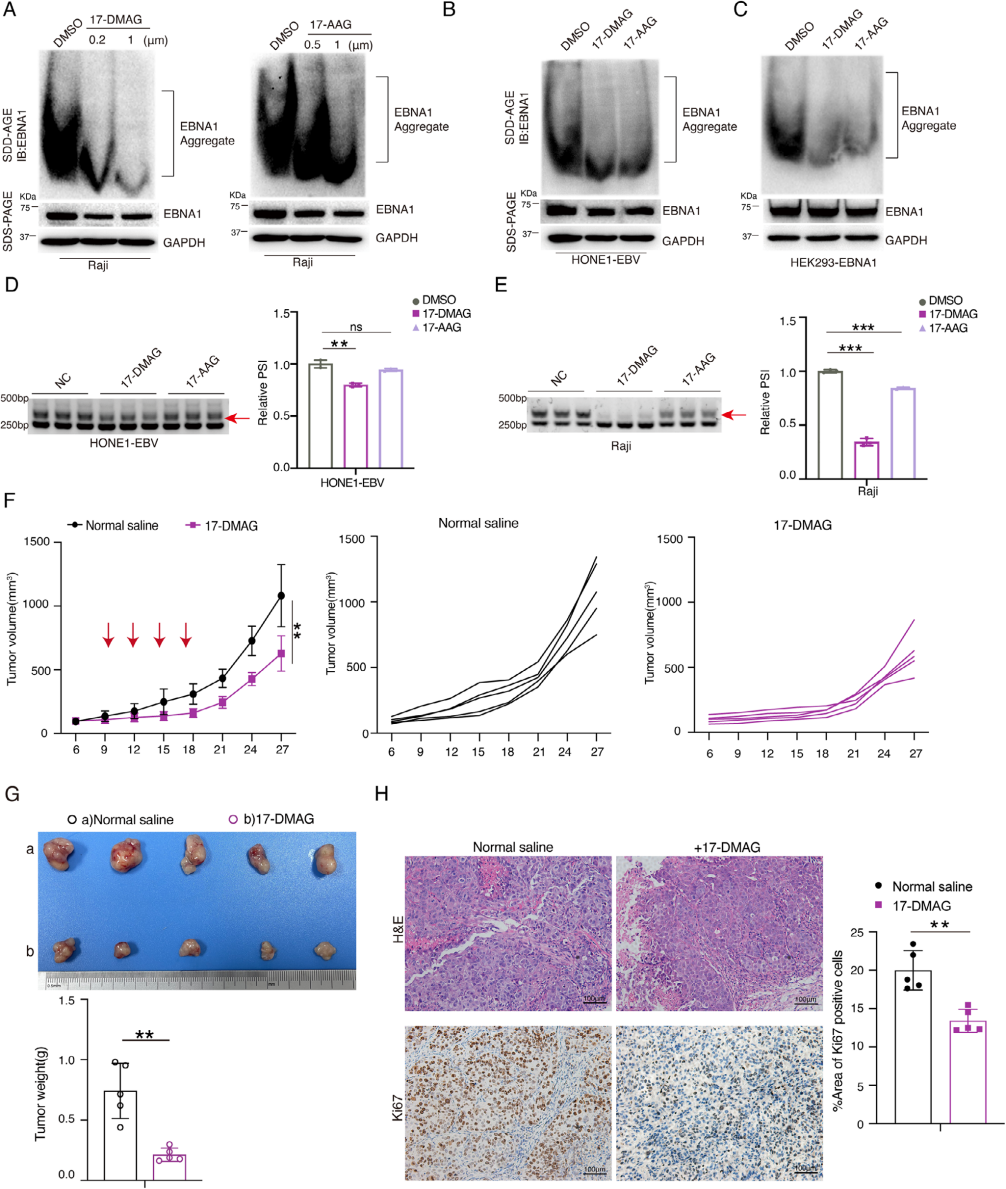
Pharmacological targeting of prion‐like domain inhibits EBNA1 protein aggregate formation, promotes SRRM1 alternative splicing and inhibits tumor progression. A–C) Raji (A), HONE1‐EBV (B), and HEK‐293‐EBNA1 (C) cells were treated with HSP90 inhibitor 17‐DMAG or 17‐AAG for 48 hours. The expression of endogenous EBNA1 prion‐like protein aggregates was detected by SDD‐AGE and SDS‐PAGE analysis. D,E) RT‐PCR and agarose gel electrophoresis experiments were performed to detect the SRRM1 alternative splice isoform in HONE1‐EBV (D) and Raji (E) cells. Red arrows indicate the position of SRRM1 splice isoforms. The relative expression values of the splice isoforms are plotted (right panel). RT‐PCR was detected on three biological replicates. F,G) HONE1‐EBV cells were implanted subcutaneously into BALB/C nude mice, and these mice were treated with 17‐DMAG (total cumulative dose 28 mg kg^−1^). Mice were randomly grouped into (a) normal saline (saline injection group) and (b) 17‐DMAG group (*n* = 5, each group). Growth curves of HONE1‐EBV xenograft tumors subcutaneously in different treatment groups are shown (F). Red arrows indicate that the administration treatment was performed at that time (F). Macroscopic images and the excised tumor weights in each group are presented (G). H) H&E staining and immunohistochemistry were performed to assess tumor tissue morphology and expression levels of Ki67. Scale bar: 100 µm. The data is shown as the Mean ± SD. **p < 0.01,***p < 0.001, ns, not significant.

A growing number of studies have shown that EBNA1 is a potential therapeutic target,^[^ ^62^ ^]^ and Sun et al. found that HSP90 inhibitors, 17‐DMAG or 17‐AAG, inhibited EBNA1 function via the Gly‐Ala‐rich repeats without affecting its stability and half‐life.^[^ ^63^ ^]^ In the above study, we demonstrated that EBNA1 prion‐like protein aggregation is determined by the Gly‐Ala‐rich repeat domain. Here, the HSP90 inhibitor provides an excellent research tool for our next experimental exploration. Indeed, 17‐DMAG or 17‐AAG significantly inhibited endogenous EBNA1 prion‐like protein aggregate formation in multiple EBV‐positive cells (**Figure** **7**A–C; Figure S6A, Supporting Information). To determine whether targeting EBNA1's prion‐like domain affects the SRRM1 exon skipping, we treated HONE1‐EBV, Raji, and B95.8 cells with 17‐DMAG and 17‐AAG for 48 h, and extracted the cellular RNA for RT‐PCR and agarose gel electrophoresis. The results showed that targeting EBNA1's prion‐like domain significantly promoted SRRM1 exon 4 skipping events in these cells, with 17‐DMAG being most effective (Figure 7D,E; Figure S6B, Supporting Information).

To further demonstrate the role of the EBV‐EBNA1 prion‐like domain in tumor development, we constructed a xenograft model in nude mice by implanting HONE1‐EBV or HK1‐EBV cells (EBV‐positive nasopharyngeal carcinoma cells) and subsequently injected 17‐DMAG, after which we monitored tumor growth. 17‐DMAG significantly inhibited the tumor growth in vivo in both the HONE1‐EBV (Figure 7F,G) and the HK1‐EBV group (Figure S6C–E, Supporting Information). Immunohistochemistry assay showed that Ki67 expression was significantly reduced by 17‐DMAG treatment (Figure 7H; Figure S6F, Supporting Information). Together, these findings demonstrate that the EBV‐EBNA1 prion‐like domain promoted nasopharyngeal cancer progression, and that targeting the EBNA1 prion‐like domain significantly reduced the progression of nasopharyngeal cancer. To further explore the pharmacotoxicity of the HSP90 inhibitors 17‐DMAG in nude mice, we weighed the mice, including their major organs (heart, liver, spleen, lungs, kidneys), after saline or 17‐DMAG injection. The results revealed that the body weight remained at a relatively stable level (Figure S7A, Supporting Information) and there was no significant changes in the weights of major organs (Figure S7B, Supporting Information). The routine blood tests in mice were analyzed and revealed that no significant difference was observed in the 17‐DMAG administration group compared with the control group (saline injection) (Figure S7C, Supporting Information). H&E staining analysis showed no inflammatory or histologic changes in heart, liver, spleen, lung, or kidney tissue following 17‐DMAG treatment (Figure S7D, Supporting Information). These results indicate that the use of 17‐DMAG is biologically safe in vivo, providing a potential for targeting the prion‐like domain of EBNA1.

## 3 Discussion

**Figure 8:**
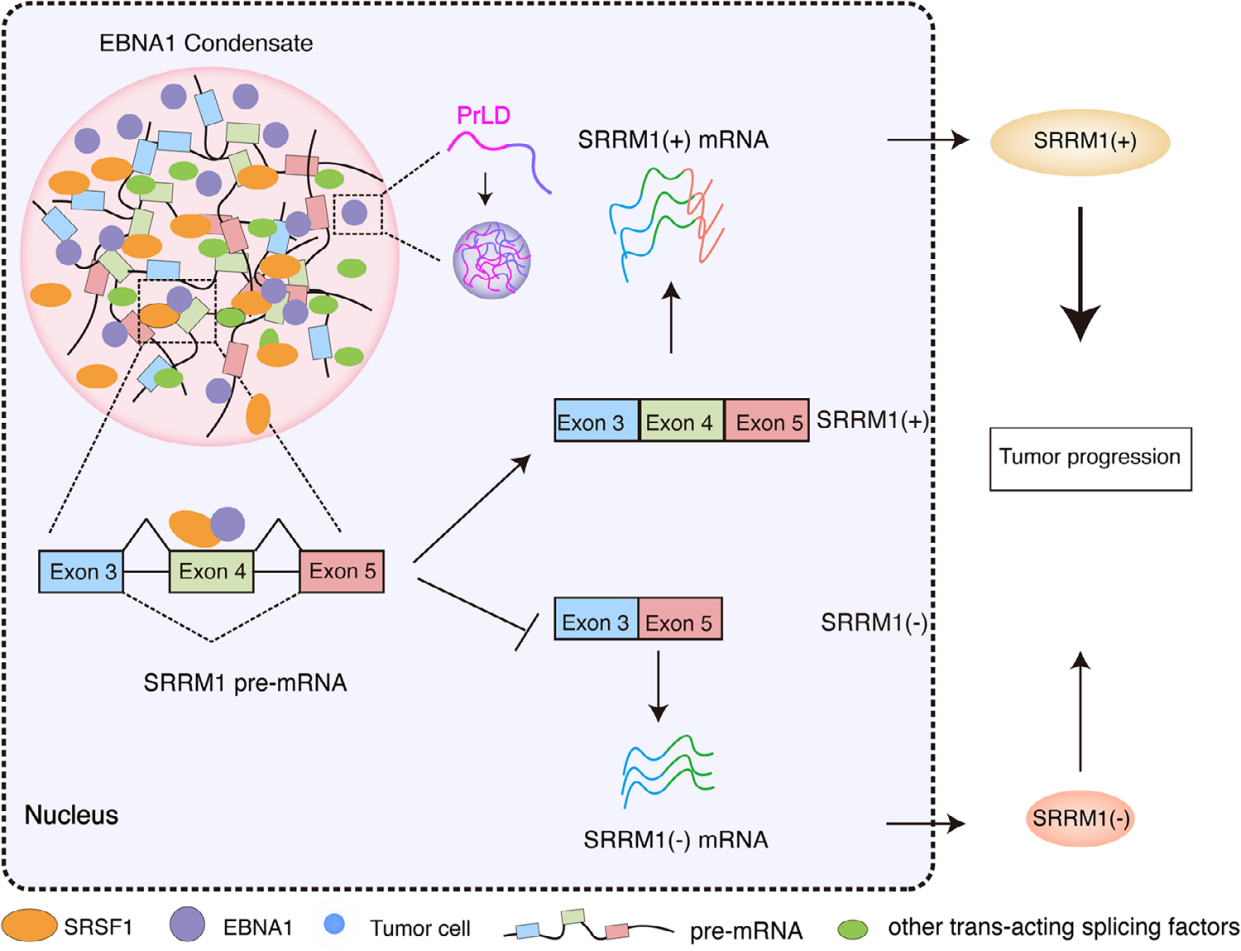
Diagram illustrating the mechanism by which EBV EBNA1 with prion‐like domains regulates the alternative splicing of SRRM1. Briefly, the prion‐like domain of EBNA1 drives protein aggregation, which in turn drives liquid–liquid phase separation. Through this mechanism, EBNA1 interacts with SRSF1 to influence the host's alternative splicing pattern. Notably, this interaction modulates the expression of the SRRM1 splicing isoforms, resulting in the production of two distinct splicing isoforms: SRRM1(+) and SRRM1(−). Intriguingly, these isoforms exhibit opposing roles in the regulation of tumor progression, highlighting the functional significance of EBNA1‐mediated splicing alterations.

In this study, we observed that EBNA1 aggregates in the nucleus and in EBV‐positive tumor tissues by means of immunogold electron microscopy and thioflavin T staining, presenting evidences that the prion‐like domain drives EBNA1 protein aggregation, an essential feature of prion‐like proteins. Deletion of the prion‐like domain of EBNA1 suppresses the formation of protein aggregates, phase separation, and the proliferation of nasopharyngeal carcinoma cells. Here, we report for the first time that EBNA1 is a prion‐like protein, verified using cell‐based assays and the *Saccharomyces cerevisiae* Sup35p prion identification system.^[^ ^21^ ^]^ In addition, we identified that EBNA1 interacts with the splicing factor SRSF1 to regulate the expression of the SRRM1 splicing isoforms, thereby promoting EBV‐associated tumor development. Treatment of EBV‐positive cancer cells with 17‐DMAG, which is capable of targeting the prion‐like domain, resulted in a significant reduction in the growth of transplanted tumors in vivo (**Figure** **8**).

Multivalent interactions mediated by low‐complexity sequences (LCS) trigger liquid–liquid phase separation (LLPS). By evaluating the phase separation propensity of 22 proteins in the FUS family, they found that Glycine‐rich regions serve as optimal spacer regions, as they confer conformational flexibility to peptide bonds, ultimately enhancing condensate fluidity.^[^ ^31^, ^64^ ^]^ For instance, the low‐complexity domain (LCD) of FUS composed of G/S[Y]G/S motifs is associated with phase transitions and the formation of hydrogels constituted by amyloid‐like fibers.^[^ ^65^ ^]^ In this study, the Gly–Ala repeats within the EBNA1 prion‐like domain (PrLD)—a typical LCD—may represent one of the contributing factors for EBNA1‐mediated LLPS. Our research team has long focused on elucidating the functions of EBNA1, a key oncoprotein encoded by EBV. During prior electrophoretic analyses, we noted anomalous EBNA1 protein aggregation patterns, prompting further investigation. Building upon bioinformatic predictions by *Tetz* et al.^[^ ^18^ ^]^ and mechanistic insights from *Xu* et al.,^[^ ^21^ ^]^ we posited two interrelated hypotheses: 1) does EBNA1 harbor a functional PrLD?; 2) could PrLD‐mediated phase separation underlie the observed aggregation? While computational algorithms (PLAAC/PAPA) identified putative PrLDs in EBNA1, the functions of PrLD in EBNA1protein remain unclear. Here, we discovered that EBNA1‐PrLD behaves a prion protein and drives phase separation of EBNA1. The discovery of the prion‐like domain may unveil a previously unrecognized molecular strategy employed by the virus. As a key factor for maintaining EBV episomes, EBNA1 forms cell cycle‐dependent DNA cross‐linking with the EBV plasmid replication origin oriP.^[^ ^66^ ^]^ Whether this process utilizes phase separation mechanisms to enrich factors involved in episome replication and segregation, thereby ensuring efficient viral genome proliferation, warrants in‐depth investigation.

Similar to other prion‐like proteins, EBNA1's PrLD enables phase separation, which is a common mechanism for creating functional compartments. PrP^C^is characterized by a large intrinsically disordered N‐terminal domain and a structured C‐terminal globular domain. The intrinsically disordered N1 domain, which is proteolytically processed into a completely disordered fragment in vivo, is both necessary and sufficient for PrP liquid–liquid phase separation, forming highly dynamic liquid droplets.^[^ ^67^ ^]^ Tau protein LLPS is primarily driven by electrostatic interactions between the negatively charged N‐terminal and positively charged C‐terminal regions.^[^ ^68^ ^]^ RNA‐binding proteins (RBPs) containing PrLDs, such as FUS and TDP‐43, provide some of the most well‐studied examples of RBPs that undergo LLPS. These proteins are found in cytoplasmic inclusions of degenerating neurons, which are key pathological hallmarks of amyotrophic lateral sclerosis and frontotemporal dementia.^[^ ^69^ ^]^ The N‐terminal domain of TDP‐43 is critical for phase separation; deletion of its disordered domain reveals that LLPS still depends on oligomerization of the N‐terminal domain.^[^ ^70^, ^71^ ^]^ Interactions between the N‐terminal PrLD and C‐terminal RNA‐binding domain of FUS are pivotal for driving its phase separation.^[^ ^31^ ^]^ The PrLDs also drive the formation of membraneless organelles.^[^ ^39^, ^72^, ^73^ ^]^ However, the biological outcomes are varied. In the case of FUS and TDP‐43, abnormal phase separation and subsequent aggregation are associated with neuronal toxicity and disease progression. Our studies show that deletion of the N‐terminal prion‐like region in EBNA1 abolishes its LLPS capacity (Figure 3). Furthermore, we aimed to validate the phase separation behavior using recombinant GFP‐tagged EBNA1‐PrLD proteins in vitro. However, these experiments faced substantial challenges, likely due to the high Gly‐Ala‐repeat sequence in the EBNA1 PrLD, which hindered successful protein purification.

Aggregation of prion‐like proteins may promote cancer progression. Overexpression of PrP^C^ promotes proliferation of different cancer cell lines, such as gastric cancer^[^ ^74^ ^]^ and pancreatic cancer.^[^ ^75^ ^]^ The EBNA1 PrLD promotes proliferation of nasopharyngeal carcinoma cells (Figure S1D,E, Supporting Information). Studies have shown that aggregation of the cancer‐associated p53 isoform Δ133p53β depends on binding to interaction partners, including p63 family members or the chaperonin containing TCP‐1 (CCT) chaperone complex.^[^ ^76^ ^]^ We found that EBNA1 condensates are enriched with multiple interacting proteins (Figure S3, Supporting Information). In this study, we investigated the mechanism by which EBNA1 interacts with SRSF1 to regulate alternative splicing of host genes, thereby promoting proliferation of nasopharyngeal carcinoma cells. P53 aggregation leads to loss of tumor suppressor function, FUS aggregation promotes neurodegenerative diseases and tumor metastasis, and YAP/TAZ condensates drive cellular stemness—these processes are all associated with imbalance of endogenous physiological regulation.^[^ ^64^, ^77^ ^]^ The low sequence complexity of the EBNA1 PrLD allows evasion of host immune surveillance in the EBV genome while maintaining stability of viral genetic material through a prion‐like phenotype, a viral adaptive strategy not possessed by cellular proteins. It is worth noting that an intermediate LLPS step is required for these processes.

The development of cancer is associated with the formation of amyloid‐like substances through protein aggregation. Increasingly, the liquid‐to‐solid transition of proteins linked to neurodegenerative diseases—such as RNA‐binding proteins TDP‐43 and FUS, as well as Tau and α‐synuclein—has emerged as a critical factor in disease pathogenesis. Notably, EBV infection is a major contributor to multiple sclerosis, with EBNA1 playing a central role in disease progression.^[^ ^12^, ^78^, ^79^ ^]^ Whether the PrLD of EBNA1 and its aggregation properties promote disease progression warrants in‐depth investigation. Designing drug candidates or small molecules to specifically target the prion‐like domain is a challenging yet achievable goal. Given the unique biophysical properties of prion‐like domains, peptides that mimic or disrupt their phase separation behavior could be developed. For instance, peptides could be designed to competitively bind to the regions of EBNA1 involved in phase separation, preventing the self‐association that leads to the formation of membrane‐less compartments. Additionally, compounds that have been shown to disrupt similar phase separation processes in other proteins, such as those involved in neurodegenerative diseases,^[^ ^80^, ^81^ ^]^ could be screened and modified for specificity against EBNA1's prion‐like domain. Natural products with anti‐aggregation properties might also hold promise, as they could potentially be adapted to target EBNA1 aggregates and prevent their formation.

Despite the progress made in understanding the role of the PrLD in EBNA1, numerous questions remain unanswered. One of the questions is the determination of the exact 3D structure of the PrLD. Knowledge of its precise architecture would provide crucial insights into how it mediates phase separation and interacts with other molecules. Another open question pertains to the regulation of the prion‐like domain's activity. How does the cell, and in particular, how does EBV control the phase separation mediated by this domain? Are there specific post‐translational modifications, such as phosphorylation or acetylation, that modulate its function? Additionally, what environmental cues, both intracellular and extracellular, trigger the onset or termination of phase separation?

In summary, we identified a PrLD in EBNA1 and observed EBNA1 protein aggregation in EBV‐positive tumors. This domain is accountable for LLPS of EBNA1 and its ability to regulate host cells alternative splicing events. The discovery of the PrLD in EBNA1 and its implications has the potential to revolutionize our understanding and treatment of EBV‐related tumors and neurodegenerative diseases.

## 4 Experimental Section

### Cell Culture and Reagents

HEK‐293 cells were cultured in Dulbecco's modified Eagle's medium (Gibco) supplemented with 10% fetal bovine serum (FBS, Biological Industries). The following cell lines were cultured in RPMI 1640 medium with 10% FBS: the normal human gastric epithelial cell line GES‐1, EBV‐negative nasopharyngeal carcinoma cell lines (HK1, HONE1, HNE1, 5–8F, 6–10B, CNE1, and CNE2), and EBV‐negative gastric cancer cell lines (AGS, SGC7901, MKN‐7, BGC‐823 and HGC27). The suspension cell lines, namely Akata (EBV‐negative Burkitt's lymphoma cell line), Raji (EBV‐positive Burkitt's lymphoma cell line), GM12878 (EBV‐positive B lymphoma cell line), B95.8 (EBV‐ transformed tamarin (Saguinus oedipus) cell line), were cultured in RPMI 1640 medium containing 10% FBS. HK1‐EBV and HONE1‐EBV (EBV‐positive nasopharyngeal carcinoma cell lines), and AGS‐EBV (EBV‐positive gastric cancer cell line) were generated by stably transfecting the EBV‐BAC plasmid into HK1, AGS, and HONE1 cells. The EBV genome derived from Akata cells was cloned into a BAC vector to construct a BAC clone named Akata‐BAC. The GET recombination system in *E. coli* enabled rapid and precise modification of the Akata‐BAC genome. Transfection of Akata‐BAC‐GFP into Akata cells formed stable EBV episomes, which generated recombinant viruses upon antibody stimulation. The recombinant viruses were used to infect EBV‐negative cells, and cell clones containing only Akata‐BAC‐GFP were screened out.^[^ ^82^ ^]^ AGS‐EBV cells were cultured in Ham's F‐12 (Gibco) medium containing 10% FBS. All cells were verified to be *Mycoplasma* negative (TaKaRa) prior to culture and were reauthenticated using the short tandem repeat Multiamplification Kit (Goldeneye DNA ID system 20A, Peoplespot) every six months. All cell lines were maintained in a humidified incubator at 37 °C under 5% CO_2_ and obtained from American Type Culture Collection. 17‐DMAG (HY‐10389) and 17‐AAG (HY‐10211) were purchased from MedChemExpress.

### Plasmids

The pcDNA3.1‐3 × Flag‐SRRM1(+), pcDNA3.1‐3 × Flag‐SRRM1(−), EGFP‐EBNA1, and EGFP‐EBNA1△90‐325 expression plasmids were constructed by performing PCR amplification on the target DNA fragments and cloning them into the empty plasmids tagged with Flag or EGFP. The MSCV‐N‐EBNA1 was purchased from Addgene (#37954). EBNA1 truncated expression plasmids were designed based on the gene sequences of different structural domains of EBNA1. EBNA1△41‐64, EBNA1△65‐89, EBNA1△90‐325, EBNA1△326‐367, EBNA1△380‐386, and EBNA1△460‐607 expression plasmids were constructed using sequences synthesized by GeneScript company (Nanjing, China). EBNA1‐PrLD‐Sup35MC and EBNA1‐△PrLD‐Sup35MC expression plasmids were constructed using sequences synthesized by GeneScript company (Nanjing, China).

### Prion‐Like Amino Acid Composition (PLAAC) Visual Analysis

To retrieve the protein sequence in FASTA format, use NCBI (https://www.ncbi.nlm.nih.gov/) or Uniprot (https://sparql.uniprot.org/). To access the PLAAC website, visit http://plaac.wi.mit.edu/. The user can specify a minimum length for prion domains (set by a textbox, by default L_core_ = 60), and can optionally use organism‐specific background AA frequencies in the HMM instead of the default S.*cerevisiae* background frequencies. These frequencies can be computed from the uploaded sequences, or selected from precomputed organism‐specific frequencies (set by a dropdown list). Then, click “Analyze”.In the visualization diagram: Top: Amino acids are color‐coded based on their enrichment log‐likelihood ratio in PrLDs, following the style of the Sequence Enrichment Visualization Tool (http://jura.wi.mit.edu/cgi‐bin/bio/draw_enrichment.pl). Outer bars indicate the results of hidden Markov model (HMM) parsing. A red line represents prion domain prediction: if the red line is above the black baseline, the region is identified as a prion domain with a high potential for phase separation. Bottom: The protein sequence is provided, with amino acid sequences in red highlighting prion‐like regions. The original version of the PLAAC algorithm is described in Alberti et al.^[^ ^22^, ^48^ ^]^

### Yeast Cell Culture and Plasmid Shuffling

Yeast cells were cultured on standard rich (YPD) and synthetic Dropout (SD) Media at 30 °C. A *Saccharomyces cerevisiae* strain LJ14 (*MATa ade1‐14 trp1‐289 his3Δ‐200 ura3‐52 leu2‐3112 SUP35::loxP p[SUP35‐URA3*] [*PSI* ^+^]) was used to perform plasmid shuffling and phenotypic assays. The standard PEG/LiAc/ssDNA transformation was used to transform plasmids (including EBNA1‐PrLD‐Sup35MC or EBNA1‐△PrLD‐Sup35MC) into LJ14.^[^ ^21^, ^83^ ^]^ The transformation products were cultured on histidine‐deficient and uracil‐deficient media for 3 days at 30 °C, then inoculated onto YPD medium supplemented with 5‐fluoroorotic acid (1 mg mL^−1^, A601555, BBI) to eliminate p[SUP35‐URA3], thereby generating strains where the EBNA1‐PrLD‐Sup35MC fusion protein replaced the function of Sup35. Once clones grew, they were resuspended in sterile water for streaking onto 1/4 YPD plates orfivefold diluted and spotted onto 1/4 YPD plates to assess phenotypes.

Shuffled yeast strains were spotted on SD/‐Ade medium to verify readthrough of the premature UGA stop codon in the ade1‐14 allele, testing each strain's ability to form heritable [*PSI^+^*] states. Readthrough of this allele in [*PSI^+^*] cells yielded two monitorable phenotypes: growth on adenine‐deficient (SD/‐Ade) medium and white colony color on complete (1/4 YPD) medium, resulting from restored adenine biosynthesis that prevented red by‐product accumulation in *[psi^−^]* cells. After plasmid shuffle, yeast ade1‐14 cells expressing PrLD‐Sup35MC failed to grow on uracil‐deficient medium but grew on histidine‐deficient medium.

### Cell Transfection

Cells were cultured for a predetermined period. The siRNAs were transfected into cells for 48 h using Lipofectamine 3000 (Invitrogen) following the manufacturer's instruction. SiRNAs targeting SRSF1were obtained from HANBIO (Shanghai, China). The siRNA sequences are listed: siNC: sense 5′‐ UUCUCCGAACGUGUCACGUTT ‐3′, antisense 5′‐ ACGUGACACGUUCGGAGAATT‐3′; siSRSF1‐1: sense 5′‐GCGACAUCGACCUCAAGAATT‐3′, antisense 5′‐ UUCUUGAGGUCGAUGUCGCTT‐3′; siSRSF1‐2: sense 5′‐GGAAAGAAGAUAUGACCUATT‐3′, antisense 5′‐UAGGUCAUAUCUUCUUUCCTT‐3′; siSRSF1‐3: sense 5′‐ GGCCCAGAAGUCCAAGUUATT, antisense 5′‐ UAACUUGGACUUCUGGGCCTT‐3′.

### Cell Migration Assay

3 × 10^4^ cancer cells were transfected with the indicated plasmids using Lipofectamine 3000 (Invitrogen), and then inoculated into transwell upper chambers (Corning) in serum‐free RPMI‐1640 medium. RPMI‐1640 medium supplemented with 20% FBS was added to the bottom chamber. The cells were cultured for 12 to 48 h, after which the culture medium in the chambers was aspirated. Subsequently, the chambers were washed three times with PBS buffer, and the residual buffer was aspirated. The chambers were fixed with 4% paraformaldehyde for 30 min at 37 °C, and then gently washed three times with 1 × PBS buffer. Subsequently, the cells were stained with 0.1% crystal violet staining solution at room temperature for 10–20 min. Five randomly selected fields of view were photographed under the microscope, and the cells were counted using the Image J software.

### Clone Formation Assay

Cancer cells were transfected with plasmids (pcDNA3.1‐3 × Flag‐NC, pcDNA3.1‐3 × Flag‐SRRM1(+) or pcDNA3.1‐3 × Flag‐SRRM1(−)), respectively, using Lipofectamine 3000, and cultured in 6‐well plates (1000 cells per well). Cells were cultured until a predetermined time. The size of the cell clones was observed under a microscope. The cells were washed three times with 1 × PBS buffer and fixed with 4% paraformaldehyde for 30 min at 37 °C. Crystal violet staining solution was added and the cells were stained for 10–20 min at room temperature. Photographs were taken and the cells were counted using the Image J.

### Cell Proliferation Assay

Cancer cells were transfected with expression plasmids (pcDNA3.1‐3 × Flag‐NC, pcDNA3.1‐3 × Flag‐SRRM1(+) or pcDNA3.1‐3 × Flag‐SRRM1(−)) using Lipofectamine 3000 and then cultured in 96‐well plates (1000 cells per well). The cells were cultured until a predetermined time, after which 10 µL of CCK8 reagent (K1018, APExBIO) were added to the 96‐well plates incubating for 3 h at 37 °C. The absorbance was measured at 450 nm using a microplate reader (ThermoFisher).

### Animal Studies

Male BALB/c nude mice were purchased from Hunan Slake Laboratory Animal Co., Ltd (Changsha, China) and housed in the Department of Laboratory Animals, Xiangya School of Medicine, Central South University under specific pathogen‐free (SPF) conditions. The mice were randomly divided into three groups: 1) empty vector (NC), 2) SRRM1(+), and 3) SRRM1(−) and were used for tumorigenicity examination. HONE1 cells (either stably expressing SRRM1(+) or SRRM1(−), or empty vector as negative control) were harvested, and then single‐cell suspensions of 3.4 × 10^6^ cells were inoculated subcutaneously into the dorsal flanks of the nude mice. Each group consisted of five mice. Tumor volume and weight were recorded every three days, and tumor size was calculated as volume (mm^3^) = (length × width^2^)/2. After 25 days, the mice were sacrificed, and tumor tissues were removed, fixed and embedded. The experimental procedures involving animals were approved by the Animal Welfare and Ethics Review Committee of the Laboratory Animal Department, Central South University (approval number: CSU‐2023‐0181) and comply with the requirements of ethical standards.

### Semidenaturing Detergent Agarose Gel Electrophoresis

Semidenaturing detergent agarose gel electrophoresis (SDD‐AGE) was performed according to a published protocol with minor modifications.^[^ ^22^, ^84^ ^]^ The cell lysates were collected in lysis buffer. Protein samples were mixed thoroughly with the loading buffer (2.5 × TAE, 2.5% SDS, 25% glycerol, 0.25% bromophenol blue), placed in a 42 °C water bath for 10 min, and then allowed to stand at room temperature for 3 min. The samples were added to a 1.5% agarose gel, and the gel was electrophoresed in the buffer (0.5 × TAE containing 0.1% SDS) at a voltage of 60 V for 60 min. After electrophoresis, the gel was electrophoresed in the transfer buffer (48 mmol L^−1^ glycine, 39 mmol L^−1^ Tris‐HCI, 0.0375% SDS, 20% methanol) for 10 min. Subsequently, the gel was transferred to a buffer (48 mmol L^−1^ glycine, 39 mmol L^−1^ Tris‐HCI, 0.0375% SDS, 20% methanol) at a constant voltage of 100 V for 60 min. After transfer, standard immunoblotting experiments were carried out. The antibodies used in this study were anti‐EBNA1 (BM1083, OriGene) and anti‐Flag (F1804, Sigma–Aldrich).

### Immunoprecipitation (IP) and Western Blotting

Cells were harvested and collected in IP lysis buffer (10 mm Tris, 1% NP‐40, 2 mm EDTA, 150 mm NaCl [pH 7.5]) containing Protease/Phosphatase Inhibitor Cocktail (B14001, B15001, Selleck). For IP, lysates were incubated with anti‐SRSF1 antibody (14902S, CST) or anti‐Rabbit IgG (BA1045, Boster) at 4 °C overnight. The protein A/G beads (B23202, Selleck) were washed with IP wash buffer for three times. Antibody‐conjugated lysates were incubated with 30 µL protein A/G beads for 2–4 h. The supernatant was discarded, and 500 µL of wash buffer was added to wash the beads twice, for 1 min each time. A mixture including loading buffer and elution buffer was added, and the mixture was boiled for 10 min. Proteins pulled off the beads were added to an SDS‐PAGE gel for electrophoresis. For mass spectrometry, the gel was washed and left in the fixative for 10 min. Subsequently, the gel was washed with purified water. It was sensitized with 0.02% sodium sulfite solution for 1 min. The gel was washed twice with purified water. The gel was immersed in 0.1% silver nitrate solution for 10 min. Developer solution was added to develop the color, and the sample was sent for mass spectrometry.

For Western blotting, the proteins were separated on a 10% gel (Biosharp, China) and transferred to a PVDF membrane (Millipore). The membranes were blocked with 5% skimmed milk for 1 h at room temperature and incubated with the primary antibody at 4 °C overnight. After washing, the membranes were incubated with the HRP‐conjugated secondary antibody for 1 h at room temperature. The membrane was washed three times with TBST for 10 min each time, and chemiluminescence was performed. The antibodies used in this study were anti‐Flag (F1804, Sigma–Aldrich), goat anti‐rabbit IgG H&L (HRP) (511203, ZEN‐Bioscience), goat anti‐mouse IgG H&L (HRP) (511103, ZEN‐Bioscience), anti‐EBNA1 (BM1083, OriGene), anti‐SRRM1(12822‐1‐AP, Proteintech), anti‐SRSF1(14902S, CST) and anti‐GAPDH (60004‐1‐Ig, Proteintech).

### Immunohistochemistry and H&E Staining

In brief, sections were sequentially deparaffinized, rehydrated, preincubated with hydrogen peroxide, blocked with goat serum, and then incubated with anti‐Ki67 antibodies (27309‐1‐AP, Proteintech) at 4 °C overnight. Subsequently, biotinylated goat anti‐rabbit IgG antibodies were added at a 1:100 dilution and incubated. Finally, the slides were incubated with HRP‐conjugated streptavidin and then developed with 3,3′‐diaminobenzidine (DAB) solution (PV‐6000D, ZSGB‐BIO). After counterstaining with hematoxylin, the sections were dehydrated and mounted.

For H&E staining, the sections were hydrated, differentiated with 1% hydrochloric acid in alcohol, stained with 0.5% eosin in alcohol solution, dehydrated and clear, and sealed with drops of neutral resin. The tissue sections were observed and photographed using an Olympus BX53 fluorescence microscope.

### Thioflavin T Staining

Tissue sections were sequentially dewaxed and hydrated, antigen repaired, and blocked for endogenous peroxidase. Next, goat serum was applied to block nonspecific binding sites. incubated with anti‐EBNA1 antibodies (BM1083, OriGene) at 4 °C overnight. Secondary antibody (Cy3 conjugated; red) (SA00009‐1, Proteintech) was added and incubated for 1 h. Subsequently, 400 mM Thioflavin T (HY‐D0218, MCE) staining solution was added to the sections and incubated in the dark for 30 min. DAPI solution (C02‐04002, Bioss) was then applied for 5 min, after which the staining solution was removed and the sections were washed with distilled water for three times, each for 3 min. Finally, a drop of fluorescence mounting medium (S302380, Dako) was applied to seal the sections. The section images were captured using a laser confocal microscope Ultra‐View Vox (UltraView Vox; Perkin‐Elmer, USA).

The tissue samples were collected from the Xiangya Hospital and the Second Xiangya Hospital of Central South University between 2017 and 2023. The study was approved by the Joint Ethics Committee of the Central South University (2021‐K022). The Health Authority and informed consent was obtained from all participants. The diagnosis of all tissue samples was confirmed by histopathological examination and blindly quantified by two pathologists (Table S1, Supporting Information).

### Mass Spectrometry Analysis

Cells were cultured with RPMI 1640 or Dulbecco's modified Eagle medium supplemented with 10% FBS. After cells were harvested, the cell lysates were collected in IP lysis buffer (10 mm Tris, 1% NP‐40, 2 mm EDTA, 150 mm NaCl [pH 7.5]) with Protease/Phosphatase Inhibitor Cocktail (B14001, B15001, Selleck). For IP, lysates were incubated with anti‐EBNA1 antibody (BM1083, OriGene) at 4 °C overnight. The protein A/G beads (B23202, Selleck) were washed with IP wash buffer for three times. Antibody‐conjugated lysates were incubated with 30 µL protein A/G beads for 24 h. Subsequently, the supernatant was discarded, and the beads were washed three times. Finally, the beads with binding complexes were boiled and subjected to SDS‐PAGE for Western blotting. After electrophoresis, immerse the gel in fixing solution for 10 min, then rinse with pure water. Sensitize the gel by incubating in 0.02% sodium sulfite solution, followed by two washes. Soak the gel in 0.1% silver nitrate solution for 10 min, then wash twice. Add developing solution until protein bands are clearly visible. Excise the targeted bands, collect them, and send the gel pieces to Jingjie PTM Biolabs (Hangzhou, China) for mass spectrometry analysis.

### Immunofluorescence Confocal Microscopy

Immunofluorescence was performed in accordance with previous studies.^[^ ^46^ ^]^ Cells were inoculated into 6‐well plates at the predicted time and fixed with 4% paraformaldehyde. Then the cells were permeabilized in 0.25% Triton X‐100 buffer for ≈10 min. The cells were washed three times with 1 × PBS buffer. Subsequently, the cells were stained with DAPI solution for 5–10 min at room temperature in the dark. The images were captured using confocal laser scanning microscope.

### Bimolecular Fluorescence Complementation

HEK‐293 cells were transfected with pBiFC‐VC‐SRSF1 and pBiFC‐VN‐EBNA1 expression vectors for 48 h and fixed with 4% paraformaldehyde. The cells were permeabilized with 0.25% Triton X‐100 for 30 min. Then, the cells were washed three times with filtered PBS buffer and stained with DAPI solution for 5–10 min. Seal the sections with a drop of fluorescence mounting medium and capture the photographs using fluorescence confocal microscopy.

### Proximity Ligation Assay

In brief, Raji cells were transfected and immobilized with 4% paraformaldehyde for 15 min. Subsequently, the cells were permeabilized in 0.25% TritonX‐100 solution and blocked at room temperature for 1 h. Anti‐EBNA1(Mouse) and anti‐SRSF1(Rabbit) antibodies were added and incubated at 37 °C for 1 h. The mixed PLUS and MINUS PLA probes were incubated. After washing, the ligase solution was added and placed at 37 °C for 30 min. Next, the amplification buffer was incubated at 37 °C for 2 h according to the manufacturer's protocol using Duolink in situ fluorescence kit (Sigma–Aldrich). The samples were stained with DAPI stain for 5 min, sealed and photographed using a laser confocal image.

### Live Imaging

Cells were inoculated in a 2‐cm glass dish and transfected with EGFP‐tagged EBNA1 plasmid. The cell culture medium was replaced with phenol red‐free medium. An LSM 900 with Airyscan 2 confocal microscope was used to observe the fluorescence expression in living cells.

### Immunogold Assay

HEK‐293 cells transfected with Flag‐tagged EBNA1 plasmids were prepared as a single cell suspension. Cell deposits were fixed by a mixture of paraformaldehyde‐glutaraldehyde fixative (4%/2.5%) and ultrathin sections (≈50–70 mm) were made. Sections were placed on 200‐mesh forceps or gold meshes, with 6–8 meshes prepared for each sample. Sections were floated in 0.1% Tween‐20/phosphate buffer (T/PB) [pH7.5] and 50 mm glycine T/PB solution for 10 min each at room temperature. Sections were immersed in T/PB blocking solution containing 2.5% BSA and 2.5% FBS, kept at a constant temperature for 30 min, and incubated with primary antibody (diluted with 1 × T/PB buffer) at 4 °C overnight. They were incubated with goat anti‐mouse IgM/Gold antibody (bs‐0368G‐Gold‐1, Bioss) for 1 h at room temperature. Sections were fixed in paraformaldehyde‐glutaraldehyde fixative (4%/2.5%) for 5 min and washed three times for 2 min each with 1× T/PB buffer. Finally, the sections were incubated with 2% uranyl acetate aqueous solution for 10 min and examined using a transmission electron microscope (HITACHI, HT7700).

### TUNEL Staining

Tissue sections were sequentially dewaxed, hydrated and incubated dropwise with proteinase K (20 µg mL^−1^) for 30 min at 37 °C in an incubator. The sections were washed three times with 1 × PBS buffer, and TUNEL staining solution was added for 1 h. Sections were slowly washed three times with 1 × PBS buffer and stained with DAPI solution for 5 –10 min at room temperature in the dark. Fluorescence mounting medium was applied to seal the sections. The images were captured using Olympus BX53 fluorescence microscope.

### Reverse Transcription‐Quantitative PCR

Reverse transcription‐quantitative PCR was carried out as described.^[^ ^85^ ^]^ Total cellular RNA was extracted with TRIzol reagent (Invitrogen). The cDNA was synthesized by the Thermo Scientific RevertAid First Strand cDNA Synthesis Kit (Thermo Fisher Scientific). Then, real‐time reverse‐transcription PCR was conducted with SYBR premix Ex TaqII Kit (Takara) by means of CFX96 Real‐Time System (Bio‐Rad). *ACTIN* was employed as an internal control, and the relative expression was calculated by means of the 2^−ΔΔCt^ method. The primer sequences for RT‐qPCR as follows: *SRSF1‐*F:5′‐CCGCAGGGAACAACGATTG‐3′; *SRSF1*‐R:5′‐GCCGTATTTGTAGAACACGTCCT‐3′; GAPDH‐F: 5′‐GGAGCGAGATCCCTCCAAAAT‐3′; *GAPDH*‐R: 5′‐GGCTGTTGTCATACTTCTCATGG‐3′; *ACTIN‐F*: 5′‐GAGCTACGAGCTGCCTGACG‐3′; *ACTIN‐R*: 5′‐GTAGTTTCGTGGATGCCACAG‐3′; *SRRM1*‐F: 5′‐AACAGGATAATCGGTTCAGCAAC‐3′; *SRRM1*‐R: 5′‐CCCAAGGATTTCCGTTACTCTTT‐3′.

### RNA Extraction, Library Construction, and Nanopore Sequencing

Cells were transfected and then collected for RNA extraction. Subsequently, total RNA was qualitatively examined using Nanodrop and Agilent 2000. The construction of long‐read sequencing for cDNA libraries was performed using the Oxford Nanopore Technologies PCR‐amplified cDNA kit (SQK‐PCS109) at Benagene Company (Wuhan, China). Specifically, full‐length cDNA libraries were constructed from poly(A)+ mRNA using the cDNA‐PCR Sequencing kit. Subsequently, the cDNA was PCR‐amplified for 13–14 cycles with barcoded adapters from the Oxford Nanopore PCR Barcoding kit (SQK‐PBK004). Finally, the 1D sequencing adapter was ligated to the cDNA before loading onto an R9.4.1 FLO PRO002 flow cell for sequencing on a PromethION platform. The raw data of Nanopore sequencing downstream data are quality controlled, and the obtained sequences are filtered for low‐quality data to obtain effective data. Based on the filtered data, the high‐quality full‐length sequences are obtained. The full‐length sequences are compared with the reference genome sequences for visualization. The RNA‐seq and raw expression files and details have been deposited in NCBI GEO under accession No. GSE285484.

### Statistical Analysis

Statistical analysis was determined using independent t test or One‐way ANOVA with GraphPad Prism.9.0 software. The experimental data were expressed as mean ± standard deviation (mean ± SD). Significance were set at *p* <0.05. All of the above analyses were carried out with *p* < 0.05 indicating statistical differences, marked with “*”; *p* < 0.01, marked with “**”; *p* < 0.001, marked with “***”; and *p* < 0.0001, marked with “****”.

## Conflict of Interest

The authors declare no conflict of interest.

## Author Contributions

X.Z., Z.L., R.Z., X.Z., C.L., Y.W., Y.W., C.O., and S.F. collected, analyzed, interpreted data; C.X., J.T., and Q.Y. performed bioinformatic, computational analyses; X.Z., H.W., Q.P., and J.M. analyzed the results, wrote the manuscript; H.N. and X.X. analyzed the yeast experiment data. H.W., Q.P., and J.M. conceived the project.

## Supporting information



Supporting Information

Supporting Information

Supplemental Movie 1

## Data Availability

The data that support the findings of this study are available in the supplementary material of this article.
