# Supplementary material for: A Prion‐Like Domain in EBV EBNA1 Promotes Phase Separation and Enables SRRM1 Splicing
Source: Adv Sci (Weinh). 2025 Jul 29;12(41):e01977. doi: 10.1002/advs.202501977 (PMC12591112; doi:10.1002/advs.202501977)
Supplement: Supplementary file 1 — Supporting Information [file ADVS-12-e01977-s003.pdf]

ADVANCED SCIENCE

A Prion-like Domain in EBV EBNA1 Promotes Phase Separation and Enables SRRM1 Splicing

Zhang et al.

Supporting Information

Figure S1 to Figure S7

Table S1. The clinicopathological parameters of tissue samples. (Table S1 was provided as a separate .xlsx file)

Movie S1. FRAP experiment of HEK-293 cells that has been transfected with EGFP-EBNA1 expression vector. Scale bar: 5µm. (Movie S1 was provided as a separate .avi file)

Original blots for this manuscript

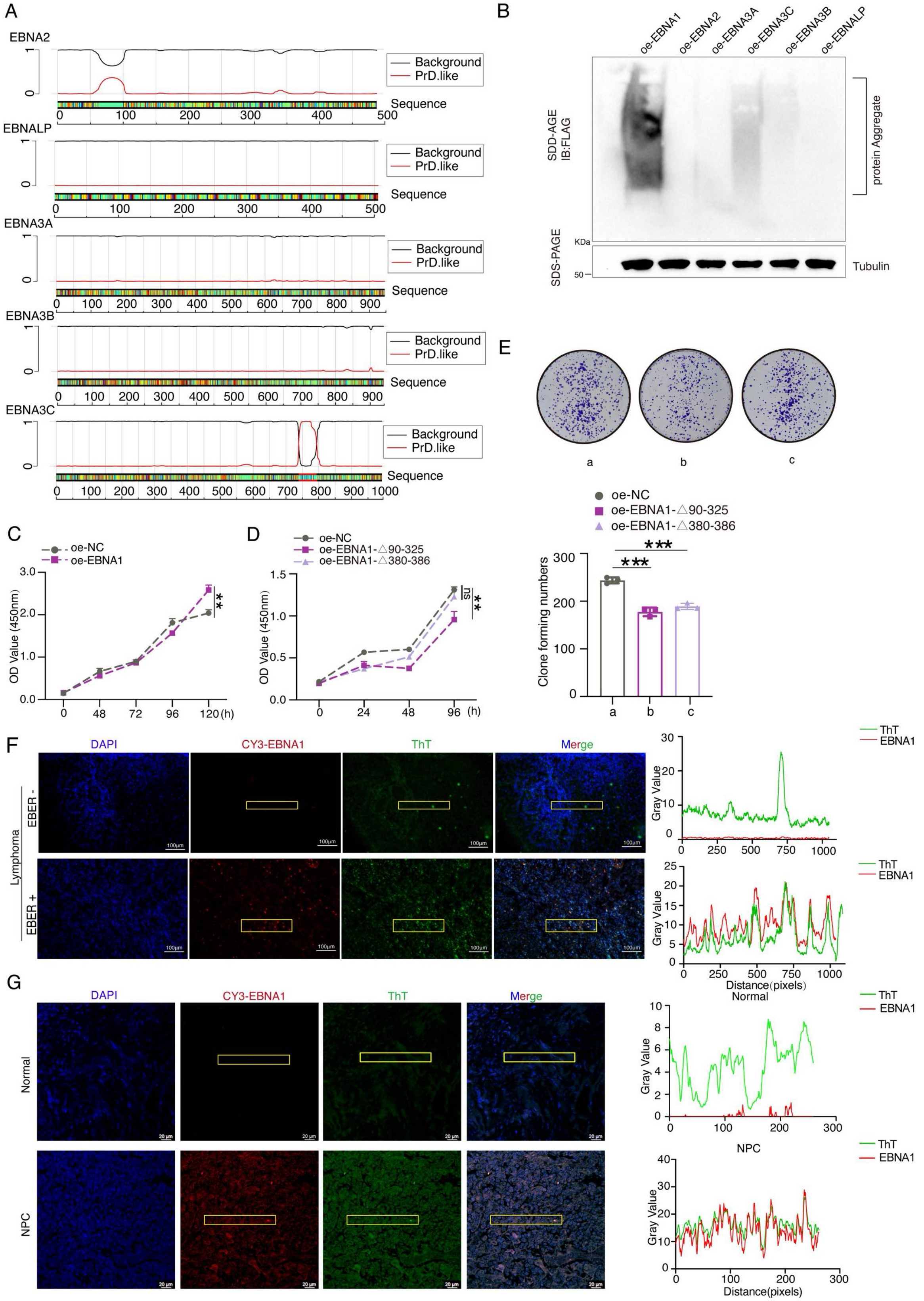

A
EBNA2
Background
PrD.like
Sequence
EBNALP
EBNA3A
EBNA3B
EBNA3C
B
oe-EBNA1
oe-EBNA2
oe-EBNA3A
oe-EBNA3C
oe-EBNA3B
oe-EBNALP
SDD-AGE IB:FLAG
protein Aggregate
SDS-PAGE
KDa
50
Tubulin
E
a
b
c
oe-NC
oe-EBNA1-△90-325
oe-EBNA1-△380-386
Clone forming numbers
C
oe-NC
oe-EBNA1
OD Value (450nm)
(h)
D
oe-NC
oe-EBNA1-△90-325
oe-EBNA1-△380-386
OD Value (450nm)
ns
(h)
F
DAPI
CY3-EBNA1
ThT
Merge
Lymphoma
EBER -
EBER +
100μm
ThT
EBNA1
Gray Value
Distance(pixels)
G
DAPI
CY3-EBNA1
ThT
Merge
Normal
NPC
20 μm
ThT
EBNA1
Normal
NPC
Gray Value
Distance(pixels)

Fig. S1. (A) Prediction of prion-like domains of EBV-encoded nuclear antigenic molecules (EBNA1, EBNA2, EBNA3A, EBNA3B, EBNA3C, and EBNALP) based on their amino acid positions and sequences was performed using the PLAAC (http://plaac.wi.mit.edu/) algorithm. **(B)** HEK-293 cells were transfected with Flag-tagged EBNA1, EBNA2, EBNA3A, EBNA3C, EBNA3B and EBNALP expression plasmids for 48 h, then, cell lysates were used to detect the EBNA1 protein aggregates by SDD-AGE and SDS-PAGE analysis. **(C-E)** HONE1 cells were transfected with indicated plasmids. Then, CCK8 assays (C, D) and clone formation assays (E) were performed to detect tumor cell proliferation abilities. **(F, G)** Co-localization of Cy3-labeled EBNA1 with thioflavin T green fluorescence in lymphoma tissues (F) or nasopharyngeal carcinoma tissues (G) was detected by immunofluorescence and thioflavin T staining assays. Scale bar: 100μm and 20μm. The data are shown as the mean ± SD. $^{**}P < 0.01$, $^{***}P < 0.001$, ns, not significant.

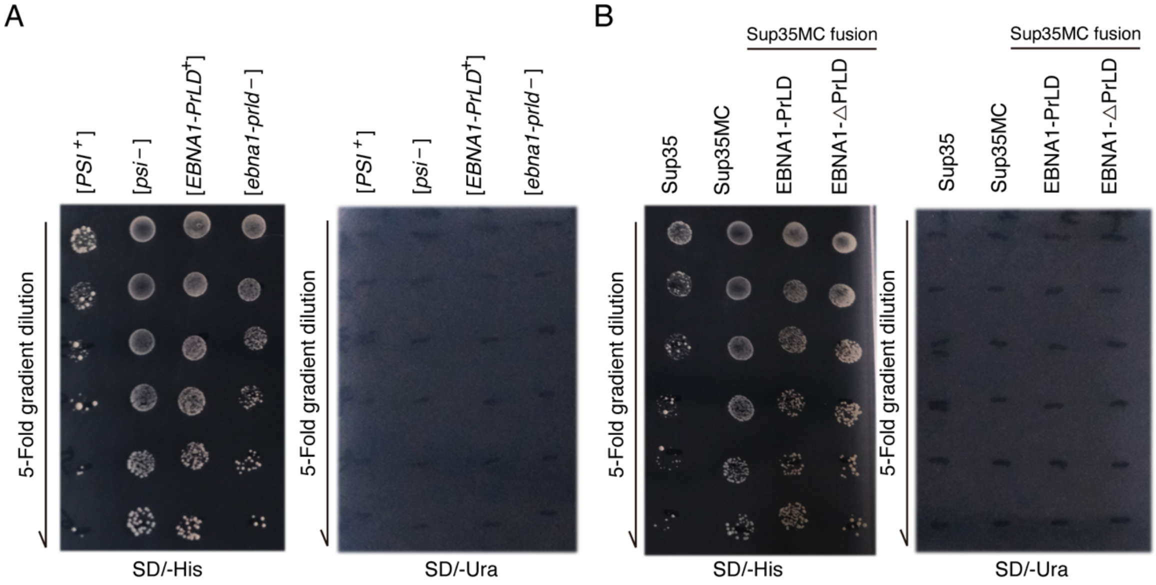

Fig. S2.(A) 5-Fold gradient dilutions of [*PSI*$^{+}$], [*psi*$^{-}$], [*EBNA1-PrLD*$^{+}$], and [*ebna1-prld*$^{-}$] stains were individually spotted onto His-deficient (SD/-His) medium and Ura-deficient (SD/-Ura) medium. **(B)** 5-Fold gradient dilutions of full-length Sup35, Sup35MC, EBNA1-PrLD-Sup35MC, and EBNA1-△PrLD-Sup35MC stains were individually spotted onto His-deficient (SD/-His) medium and Ura-deficient (SD/-Ura) medium.

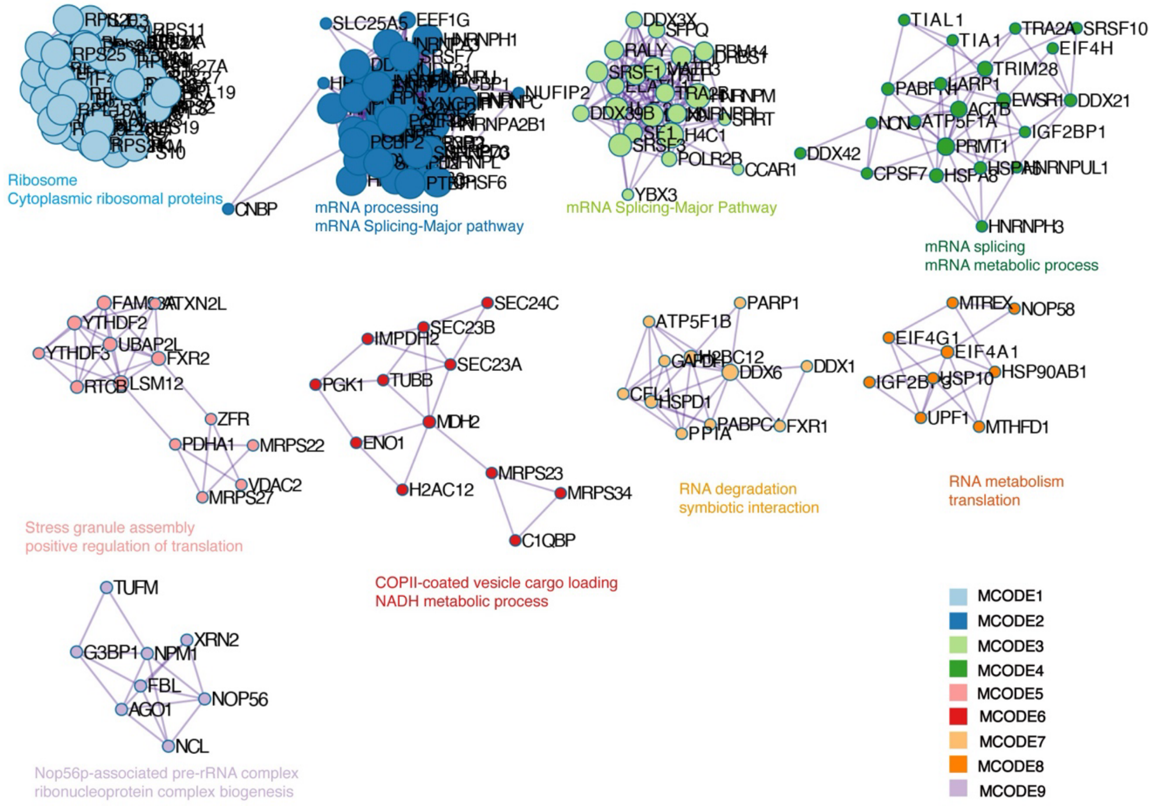

Fig. S3. Metascape software (http://metascape.org/) identified nine Molecular Complex Detection (MCODE) clusters among the 295 EBNA1-interacted proteins. The MCODE algorithm was used to cluster the Protein-Protein Interaction (PPI) network and identify potential protein complexes.

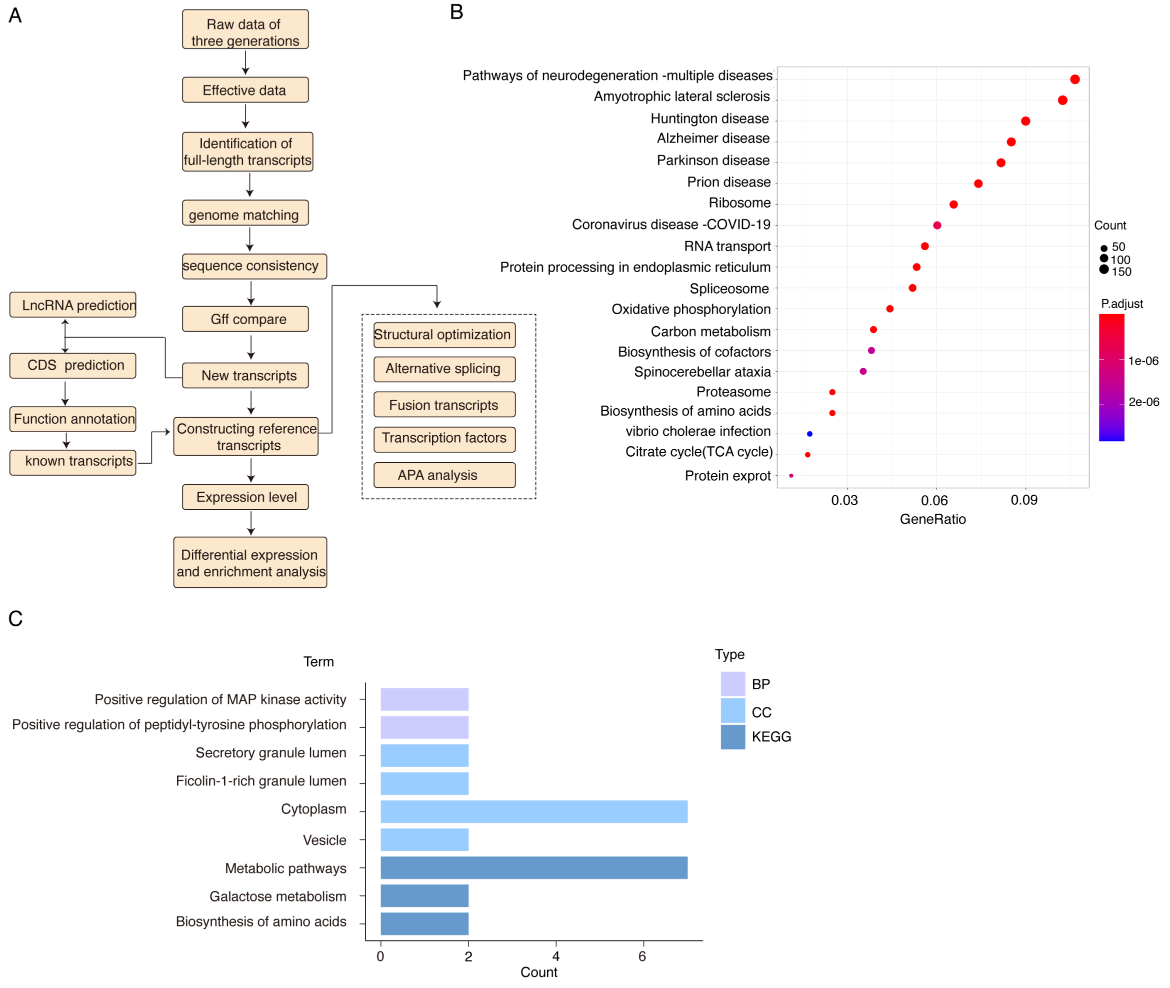

Fig. S4. (A) HEK-293 cells were transfected with either EBNA1 plasmid or empty plasmid (NC) for 48 h, then, cellular RNAs were extracted for three-generation sequencing. The flowchart of sequencing and analysis is shown. **(B)** KEGG analysis of signaling pathways associated with differentially alternative splice genes regulated by EBNA1. **(C)** KEGG analysis of EBNA1-regulated differentially expressed genes.

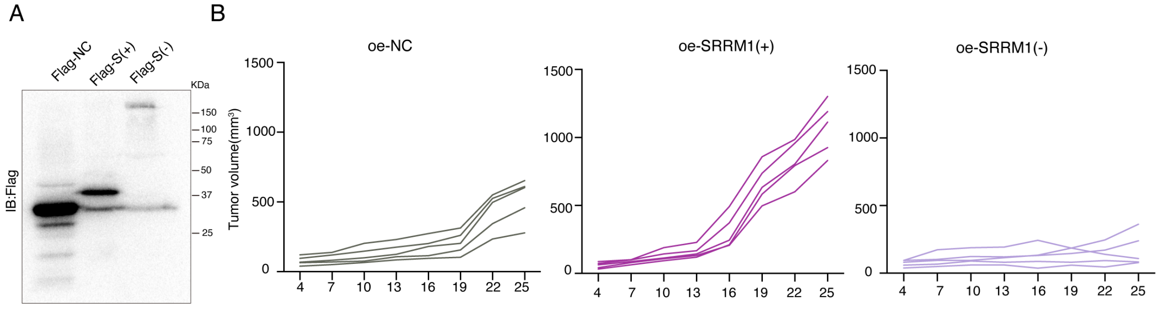

Fig. S5. **(A)** HEK-293 cells were transfected with Flag-SRRM1(+) or Flag-SRRM1(-) plasmids, respectively, for 48 h. The cell lysates were then used for Western blotting. **(B)** HONE1 cells stably transfected with Flag-NC, Flag-SRRM1(+) and Flag-SRRM1(-) plasmids were subcutaneously implanted into BALB/c nude mice to establish a xenograft growth model. The growth curves of xenograft tumors in different groups were shown (*n*=5, per group).

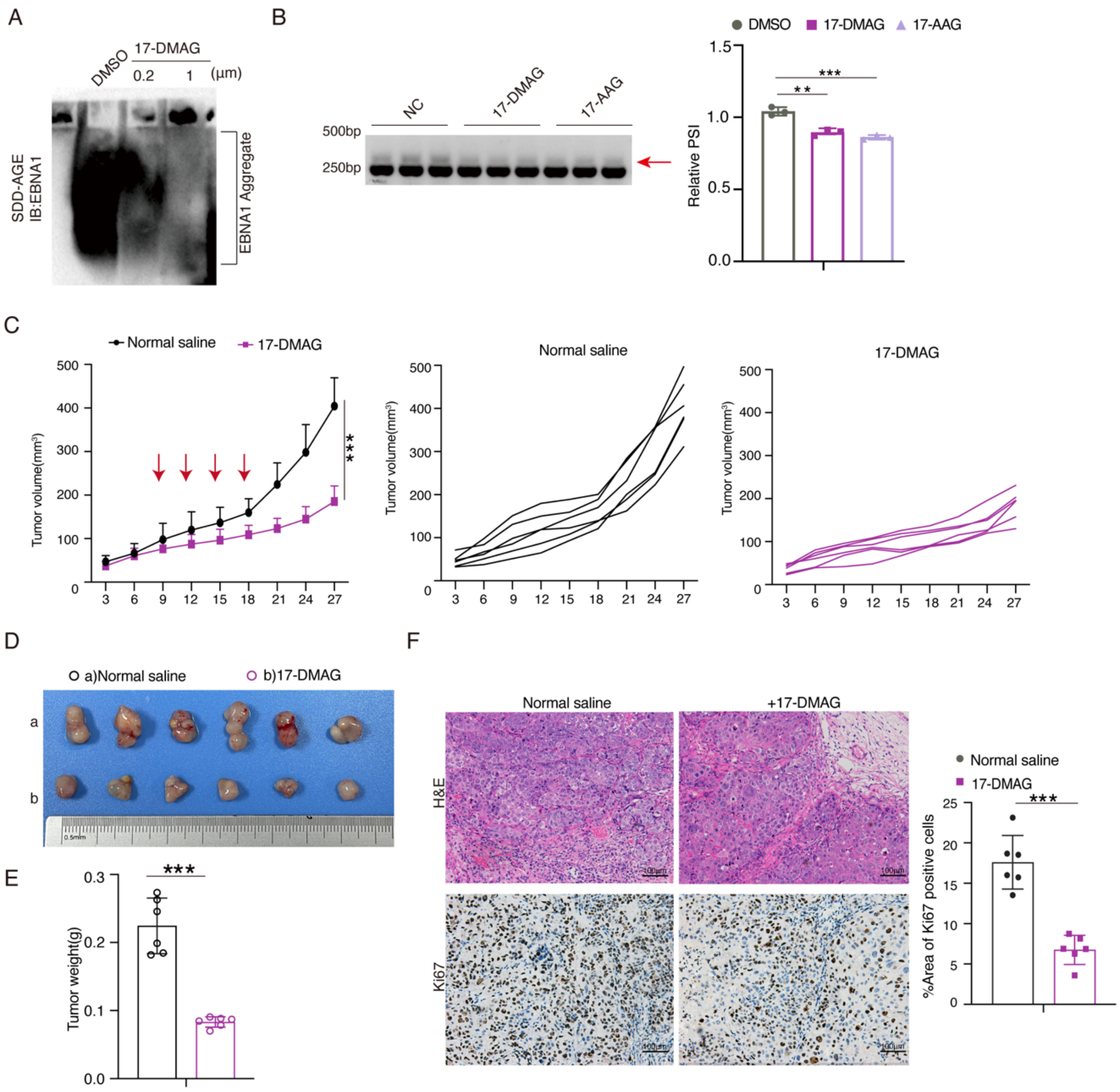

Fig. S6. (A) B95.8 cells were treated with HSP90 inhibitor 17-DMAG for 48 h. Cell lysates were used to detect the EBNA1 protein aggregates by SDD-AGE analysis. **(B)** B95.8 cells were treated with 17-DMAG (1μM) or 17-AAG (1μM) for 48 h. RT-PCR and agarose gel electrophoresis experiments were performed to detect the SRRM1 alternative splice isoform. Red arrows indicate the position of SRRM1 splice isoforms. **(C-E)** 3.5 $\times 10^6$ HK1-EBV cells were implanted subcutaneously into BALB/C nude mice, and these mice were treated with 17-DMAG (total cumulative dose 28 mg/kg). Nude mice were randomly grouped into (a) normal saline group (saline injection group) and (b) 17-DMAG group (n=6, each group). Growth curves of HK1-EBV xenograft tumors subcutaneously in different treatment groups are shown. Red arrows indicate that the administration treatment was performed at that time. Growth curves of subcutaneous tumors per nude mouse in each group are shown (C). Macroscopic images (D) and the excised tumor weights in each group are presented (E). **(F)** H&E staining and immunohistochemistry to assess tumor tissue morphology and expression levels of Ki67. Scale bar: 100μm. The data are shown as the Mean ± SD. $^{**}P < 0.01$, $^{***}P < 0.001$.

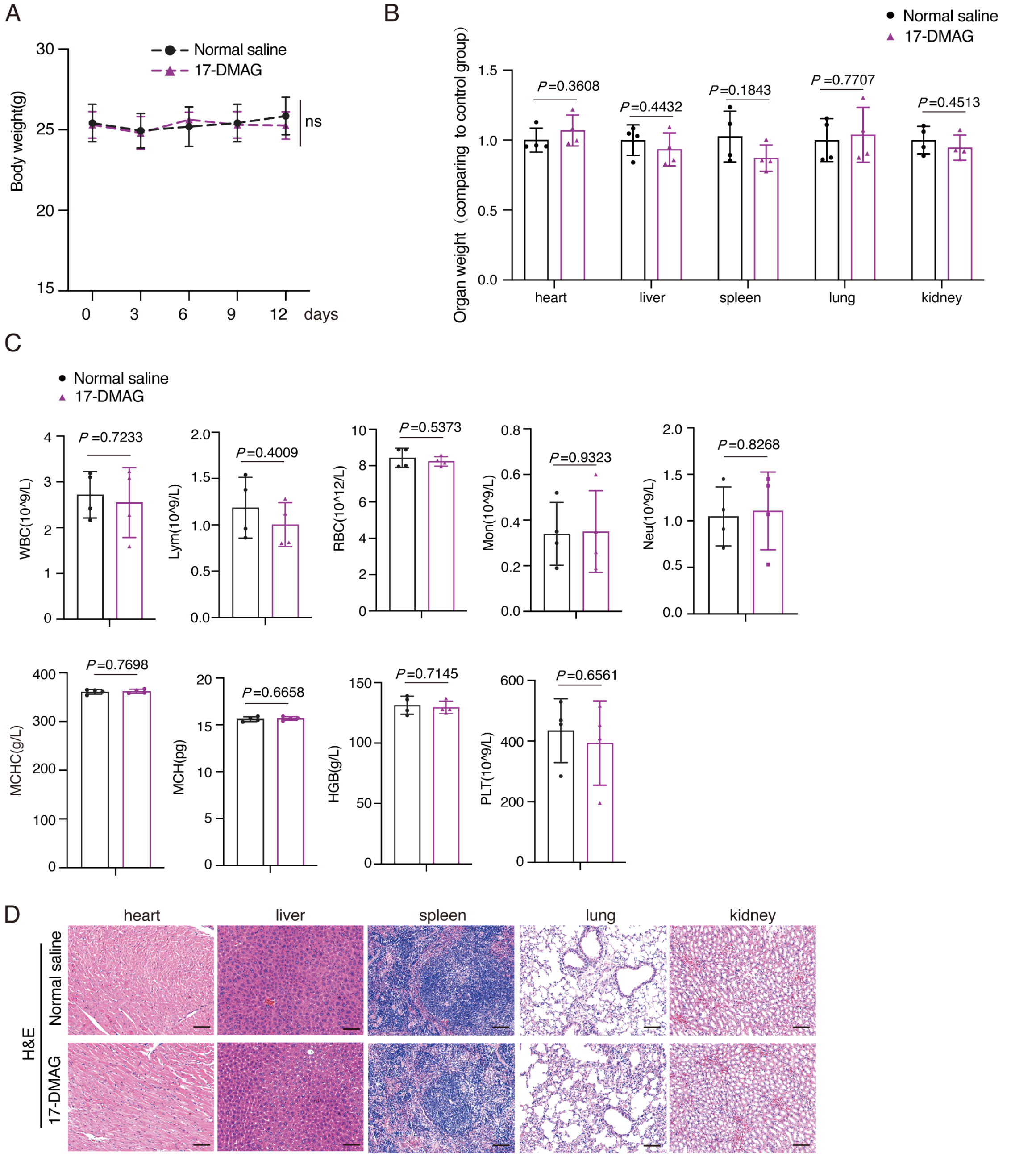

Fig. S7. (A-D) BALB/C nude mice were randomly divided into two groups: the normal saline group (saline injection group) and the 17-DMAG group (n=4, each group). The mice were injected intraperitoneally with 17-DMAG and weighed every two days, and were administered a total of four times at a cumulative dose of 28 mg/kg. The blood was collected from the eyeballs of the mice on the 12th day for routine blood tests, and the major organs (heart, liver, spleen, lungs and kidneys) were also removed for weighing and measurement. The body weight statistics (A), major organ weight statistics (B), and routine blood tests of nude mice (C) are shown. (D) H&E staining was used to assess the morphological and structural changes of major organs in mice after treatment with the HSP90 inhibitor 17-DMAG. Scale bar: 100μm. ns, not significant. MCHC,Mean Corpuscular Hemoglobin Concentration; MCH, Mean Corpuscular Hemoglobin; HGB, Hemoglobin; PLT, Platelet Count.
